# Recent advances in 3D printable conductive hydrogel inks for neural engineering

**DOI:** 10.1186/s40580-023-00389-z

**Published:** 2023-09-07

**Authors:** Sung Dong Kim, Kyoungryong Kim, Mikyung Shin

**Affiliations:** 1 Department of Biomedical Engineering, Sungkyunkwan University, Suwon, 16419 Republic of Korea; 2 Center for Neuroscience Imaging Research, Institute for Basic Science (IBS), Suwon, 16419 Republic of Korea; 3 Department of Intelligent Precision Healthcare Convergence, Sungkyunkwan University, Suwon, 16419 Republic of Korea

**Keywords:** Conductive hydrogels, 3D printing, Neural engineering

## Abstract

Recently, the 3D printing of conductive hydrogels has undergone remarkable advances in the fabrication of complex and functional structures. In the field of neural engineering, an increasing number of reports have been published on tissue engineering and bioelectronic approaches over the last few years. The convergence of 3D printing methods and electrically conducting hydrogels may create new clinical and therapeutic possibilities for precision regenerative medicine and implants. In this review, we summarize (i) advancements in preparation strategies for conductive materials, (ii) various printing techniques enabling the fabrication of electroconductive hydrogels, (iii) the required physicochemical properties of the printed constructs, (iv) their applications in bioelectronics and tissue regeneration for neural engineering, and (v) unconventional approaches and outlooks for the 3D printing of conductive hydrogels. This review provides technical insights into 3D printable conductive hydrogels and encompasses recent developments, specifically over the last few years of research in the neural engineering field.

## Introduction

Versatile devices and materials have been developed for the precise diagnosis and treatment of neurological diseases, such as neurodegenerative disorders (e.g., Parkinson’s disease and Alzheimer’s disease), neuromuscular diseases, and spinal cord and peripheral nerve injuries [1–5]. Considering the electrophysiological functions of the brain and nerve tissues, electrically conductive materials, such as metals, carbon materials, and conductive polymers, have been widely used to provide tissue-mimetic environments to cells and effectively stimulate or monitor signals from tissues [4–8]. However, most conductive materials have mechanical stiffness, low hydrophilicity and weak tissue adhesion, which are incompatible with soft nervous tissues [9, 10]. For instance, their high stiffness mismatching to soft biological tissues can cause acute inflammation and serious fibrosis upon in vivo implantation of the materials, leading to tissue engineering failures [11]. In addition to that, the neuronal cells cannot be favorably grown or differentiated on such stiff and conductive materials. The cells respond and adjust to their microenvironment, and their differentiation is strongly favored on the scaffold materials with brain-like soft modulus of 100–500 Pa [12] where cellular focal adhesion and the length of neurites increase [13]. Therefore, to resolve the physicochemical mismatch of conductive materials, tissue-mimetic hydrogels with a large amount of water can be combined with them. Such conductive hydrogels have been continuously developed for electroactive tissue engineering, tissue repair, stimuli-responsive drug delivery platforms, tissue interfacing, soft bioelectronics/robotics, and wearable/implantable sensors, owing to their tunable electrical conductivity and tissue-like mechanical properties [9, 10, 14–21].

Meanwhile, along with recent developments in precision medicine approaches, 3D printing techniques such as laser-based systems through the photopolymerization pathway, nozzle-/syringe-based extrusion systems, and jetting-based systems have been extensively used in the biomedical field over the past 10 years [22–24]. Representative examples are the 3D/4D printing of biodegradable scaffolds (e.g., polycaprolactone) for post-seeding of cells [25–29] and 3D bioprinting with cell-encapsulated inks [30–32]. To date, most printing approaches have focused on tissue engineering to fabricate “artificial organs” with personalized sizes and dimensions [33, 34]. In the next biomedical revolution, such printing techniques can be applied to the manufacturing of bioelectronics, electric circuit boards, robotics, and biosensors [35–39]. Therefore, conductive hydrogels are good candidates for ink materials that can be implanted in vivo. Nevertheless, challenges remain for the use of conductive hydrogels as printable inks with high shape fidelity owing to the requirements for the mechanical or chemical properties of the materials for numerous printing techniques. Although extensive research has been conducted on “3D printing” and “electrically conductive hydrogels” separately, the combined approaches of both have only increased recently.

In this comprehensive review, we summarize (i) advances in the preparation strategies of conductive hydrogels, (ii) various 3D printing techniques enabling the fabrication of electroconductive objects, (iii) the physicochemical properties of the conductive hydrogel inks required, (iv) their applications to bioelectronics and tissue regeneration for neural engineering, and (v) outlook to progress in printing of conductive hydrogel.

## Conductive hydrogel fabrication

Hydrogels that have higher electrical conductivity than normal hydrogels are typically considered conductive hydrogels. Normal hydrogels also have electrical conductivity owing to their tissue-like ion-rich environment [9]. Intrinsically conductive materials have been commonly used to establish hydrogel matrix with high conductivity. The addition of conductive materials to the hydrogel matrix generates additional and efficient conductive pathways to transfer electrical signals through the hydrogel matrix and consequently enhance the conductivity of the hydrogel matrix.

In this section, conductive hydrogel fabrication approaches using conductive materials and percolation strategies to achieve higher conductivity are discussed.

### Conductive filler additive manufacturing

The addition of conductive materials, such as metals and conductive polymers, to the hydrogel matrix typically improves its conductivity. Herein, we discuss representative conductive materials to be embedded and additional methods to effectively fabricate conductive hydrogels.

#### Metals

**Fig. 1:**
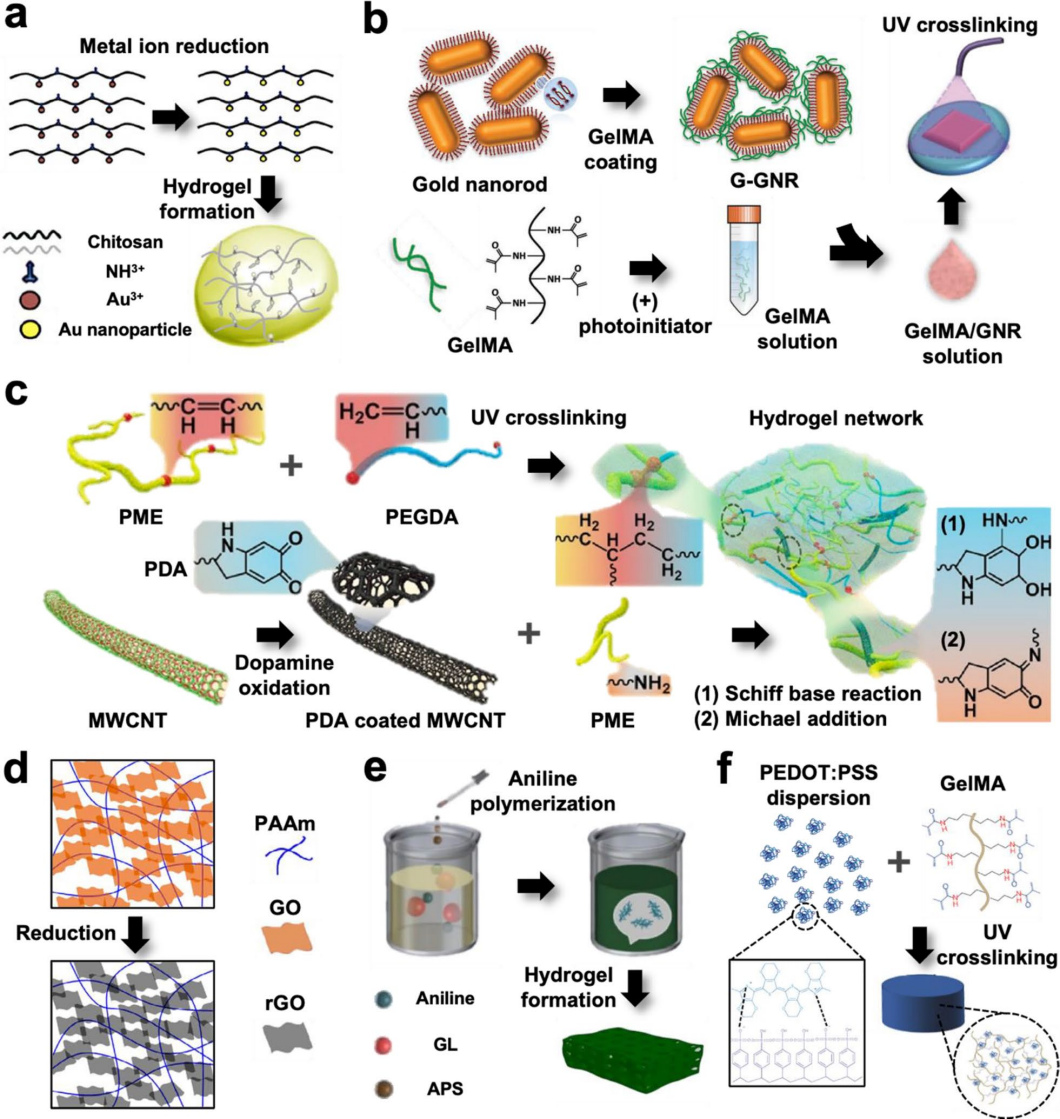
Illustration of various conductive hydrogel fabrication strategies by the addition of conductive materials. **a** Addition of metal nanoparticles using in situ metal ion reduction. Reproduced with permission from [45], copyright Elsevier, 2016. **b** Gold nanorod (GNR) addition using surface coating. Gelatin methacryloyl (GelMA)-coated GNR (G-GNR) has a colloidal stability and can be added to a photocurable GelMA solution. Reproduced with permission from [41], copyright John Wiley and Sons, 2017. **c** Addition of multi-walled CNT (MWCNT) by using surface-functionalized CNT. Polydopamine (PDA)-coated MWCNT is soluble and mixable with photocurable poly(citrate-maleic)-ε-polylysine (PME) and poly(ethylene glycol) diacrylate (PEGDA) solution. In addition, PDA coated MWCNT can be stabilized with the addition of PME. Reproduced with permission from [58], copyright Elsevier, 2022. **d** Conductive hydrogel fabrication using GO. GO can be mixed with a precursor solution and dispersed in a polyacrylamide (PAAm) hydrogel network. Reduction can be achieved after crosslinking to improve the conductivity. Reproduced with permission from [57], copyright Elsevier, 2017. **e** Conductive polymer addition using in situ monomer polymerization. The aniline monomer can be mixed with a glycyrrhizic acid (GL) solution, and ammonium persulfate (APS) causes the in-situ generation of conductive PANI in a hydrogel matrix through aniline polymerization. Reproduced with permission from [76], copyright American Chemical Society, 2022. **f** Direct addition of the conductive polymer to the precursor solution. PEDOT:PSS can be dispersed in aqueous solution and easily mixed with photocurable GelMA solution. Reproduced with permission from [78], copyright American Chemical Society, 2018

Metals are widely used because of their high conductivity and can be embedded into the polymeric network to fabricate conductive hydrogels. Gold [15, 40–45] is the most commonly used metal owing to its high biocompatibility and electrochemical stability under physiological conditions, and other metals that are used are silver [44], iron [44], eutectic gallium indium (EGaIn) [46, 47], and 2D transition metals (e.g., MXene and MoS_2_) [48, 49]. Metals can be incorporated into a hydrogel matrix in two ways. The first method is the “in situ” generation of nanoparticles from metal ions (Fig. 1a). Metal ions are prepared separately using metallic compounds such as AgNO_3_, HAuCl_4_, and FeCl_3_ and incorporated into the hydrogel through swelling [43] or mixed with a hydrogel precursor solution [44, 45]. After incorporation, the metal ions are reduced by the addition of a reducing agent [43–45] or backbone polymers that are served as reducing agents [50], forming nanoparticles in the hydrogel. The second method is simple mixing of complete metal nanomaterials, such as nanoparticles, nanowires, and nanosheets, generated using different physical and chemical methods, such as electrochemical changes, chemical reduction, and photochemical reduction [51]. Typically, metal nanomaterials are mixed with a hydrogel precursor solution and crosslinked to form a stable hydrogel (Fig. 1b) [15, 40–42, 46–49]. Thus, the colloidal stability of metal nanomaterials is essential and requires an additional coating layer (Fig. 1b) [15, 40–42, 47]. In addition, the zeta potential of nanomaterials and hydrogel backbone polymers affect the dispersion of nanomaterials in the precursor solution and the electrical stability after crosslinking. The metal fraction plays a critical role in the entire crosslinking of conductive hydrogels. In general, a higher fraction of metal not only enhances the conductivity but also increases the viscosity of the solution and the modulus of the hydrogel [40–42, 49]. Additionally, other properties, such as pore size and gelation kinetics, can be differed by adding the metals [42, 49].

#### Carbon-based materials

Carbon-based materials such as carbon nanotubes (CNTs) and graphene are synthetic materials from natural graphite. They are of interest for fabricating conductive hydrogels because of their high conductivity and stability in wet environments. However, the hydrophobic nature of CNTs and graphene is the main problem in the fabrication of hydrogel composites because they are insoluble and agglomerate in water. The presence of functional groups such as carboxyl groups on the surfaces of CNTs and graphene makes them soluble in water and provides colloidal stability during fabrication; therefore, surface-functionalized CNTs and graphene oxide (GO) are typically used to fabricate conductive hydrogels [52–57]. In addition, other CNT-stabilizing methods using a polydopamine coating (Fig. 1c) [58–60], amphiphilic cellulose nanocrystals [61], and silk sericin [62] have been reported. Thus, a uniformly dispersed carbon-based material stably contributes to electrical conduction through the hydrogel matrix. Additionally, the variation in the CNT conductivity after functionalization has not been particularly considered; however, GO has a relatively lower conductivity than graphene. Thus, a reducing process after gel formation [54, 57] (e.g., use of reduced GO (rGO) instead of GO) [55] has been used to increase hydrogel conductivity (Fig. 1d). A high fraction of carbon-based materials results in conductive hydrogels with high conductivity and mechanical strength [53–58, 61–65], which also increases the viscosity of the precursor solution [56, 65]. However, such high contents of carbon-based materials can potentially show high cytotoxicity. According to many reports regarding *in vitro* studies using carbon materials, they easily generate reactive oxygen species (ROS) depending on concentration and treatment time [66, 67]. To decrease a potential toxicity of the carbon materials with high concentrations (e.g., higher than 10 μg/mL), a biocompatible surface modification strategy, such as typical PEGylation, can be adopted [68, 69]. In addition, the shape and aspect ratio of the carbon materials are importantly considered for in vivo implantation. Chong *et al.* have reported that graphene quantum dots—which have small size of 3 nm—show much better biocompatibility than GO in mice model [70]. Poland *et al.* have also demonstrated that long multi-walled CNTs with the length of longer than 20 µm are not favorably phagocytized and accumulated in the biological tissues when compared to that of short ones, leading to less toxicity [71]. Taken together, ideal design of the conductive hydrogels with carbon materials requires consideration of versatile variables such as a type of carbon materials, shapes, aspect ratio, and surface functional groups for further biomedical applications.

#### Conductive polymers

Conductive polymers are π-bond-rich polymers, including polypyrrole (PPy), polyaniline (PANI), and poly(3,4-ethlyenedioxythiophene) (PEDOT), that exhibit electrochemical stability in physiological environments. In addition, electrons can be transferred along a polymer chain by the delocalization of π-bonded electrons, and this chain-dependent conduction preserves the conducting properties while utilizing versatile materials. Therefore, conductive polymers are commonly used in the fabrication of conductive hydrogels. Two methods can be used to add conductive polymers to the hydrogel matrix: in situ polymerization of a monomer [72–76] and mixing after stabilization using an additional dopant [77–85]. Conductive polymers are hydrophobic materials; therefore, they form large aggregates and are difficult to disperse homogeneously in hydrogels. Thus, monomers such as pyrrole and aniline are typically dispersed in the hydrogel matrix and polymerized through oxidant treatment (e.g., FeCl_3_ and ammonium persulfate) after hydrogel crosslinking (Fig. 1e) [72–74]. Otherwise, conductive polymers can be stabilized in an aqueous solution after doping. Poly(styrenesulfonate) (PSS)-doped PEDOT (PEDOT:PSS) is a representative doped form, and other doping methods, such as dopamine-doped PPy, have been reported [83]. These stabilized conductive polymers are easily mixed with the hydrogel precursor solution and used to fabricate conductive hydrogels (Fig. 1f) [77–85]. Conductive polymer additives form interconnected conductive pathways in the hydrogel matrix, enabling the transfer of electrons through it. Similar to metal- and carbon-based additives, the fraction of conductive polymers plays a critical role in the electrical and mechanical properties of conductive hydrogels, and higher fractions increase the conductivity, modulus, and viscosity.

### Conductive network formation

Although the addition of conductive materials provides a certain degree of electrical properties to the hydrogels, they still exhibit a much lower conductivity (< 1 S/m) than the original conductive materials (> 100 S/m). This is because numerous small isolated conductive domains covered by an insulating domain are formed in the hydrogels, inhibiting charge transfer over the matrix. Herein, we discuss a fabrication strategy to form a well-connected percolating conductive network.

#### Pure conductive polymer hydrogel

Fabricating conductive hydrogels with only conductive polymers minimizes the fraction of insulating domains and enhances the continuity of the conductive domains in the hydrogel matrix. However, conductive polymers are typically difficult to use as hydrogel backbone polymers because of their hydrophobic nature. They form agglomerations instead of uniform networks; therefore, hydrogels with high water content and low modulus are difficult to be achieved.

**Fig. 2:**
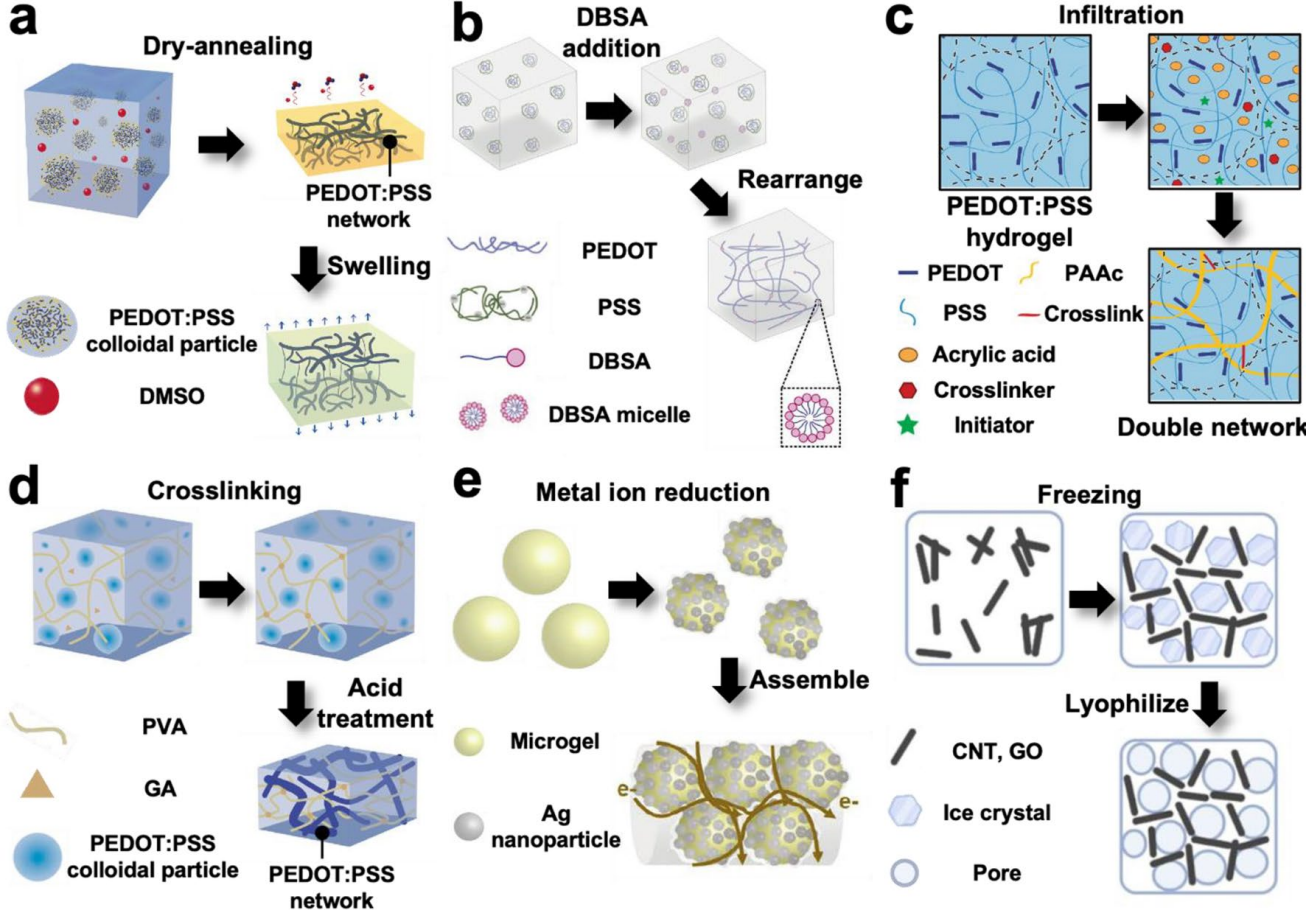
Illustration of conductive network formation strategies for high conductivities. a, b) Pure conductive polymer hydrogel fabrication using an additive. **a** DMSO addition supports formation of physically interconnected PEDOT:PSS network during dry-annealing. Reproduced with permission from [88], copyright Springer Nature, 2020. **b** DBSA micelle addition causes rearrangement of PEDOT:PSS colloidal particles and formation of a physically interconnected conductive hydrogel network within few minutes. Reproduced with permission from [91], copyright John Wiley and Sons, 2019. **c** Percolating conductive network hydrogel fabrication from a pure conductive polymer hydrogel. The monomer, crosslinker, and initiator can be infiltrated into the pure conductive polymer hydrogel and cause secondary network formation while maintaining primary percolating conductive network. Reproduced with permission from [93], copyright Springer Nature, 2018. **d** Fabrication of a percolating conductive network hydrogel from non-conductive hydrogel. PEDOT:PSS can be dispersed in the precursor solution. After crosslinked PVA network formation using glutaraldehyde (GA), acid treatment causes the rearrangement of PEDOT:PSS colloidal particles and formation of a secondary percolating conductive network. Reproduced with permission from [99], copyright John Wiley and Sons, 2022. **e** Spatial separation of conductive nanocomposite using microgel. Ag nanoparticles are only produced on the surface of the microgel through in situ reduction and a densely packed Ag nanoparticle percolating network is produced after microgel assembly. Reproduced with permission from [100], copyright John Wiley and Sons, 2019. **f** Spatial separation of conductive nanocomposite using freezing. CNT and GO are located in the surrounding region of ice crystal during freezing. This improves the percolating conductive network and can be maintained by crosslinking at ambient conditions. Reproduced with permission from [102], copyright John Wiley and Sons, 2022

Recently, various pure conductive polymer hydrogel fabrication methods have been reported using PEDOT:PSS with additives such as acids [86], ionic liquids [87], and secondary dopants (e.g., dimethyl sulfoxide (DMSO) [88–90], ethylene glycol [90], and 4-dodecylbenzenesulfonic acid (DBSA) [91, 92]). Frequently, the additive support conformation changes from PEDOT:PSS colloidal particles to physically interconnected networks during the dry-annealing process, and more interconnections between the PEDOT polymers are generated than in the process without additives (Fig. 2a). After washing and re-swelling this PEDOT:PSS film, PEDOT:PSS hydrogels with high stability and conductivity are formed [86–90]. In contrast, PEDOT:PSS films without additives exhibit fragmentation [88] and conductive path failure because of gap generation after swelling [87]. Additionally, an increase in the PEDOT to PSS ratio by the removal of PSS, which means a decrease in the insulating domain in the hydrogel matrix when using acid and ionic liquids as additives, has been reported [86, 87]. The concentration of the additive significantly affects the hydrogel properties, and an optimization process is required. After the optimization process, the PEDOT:PSS hydrogel frequently exhibits ultrahigh conductivity (conductivity > 800 S/m), high water content (> 80 wt%), and low modulus (< 5 MPa for DMSO and < 50 kPa for others) [86–90]. Moreover, in situ conductive hydrogel fabrication can be conducted using DBSA as an additive, which is useful for injection and printing (Fig. 2b) [91, 92].

#### Percolating conductive nanocomposite network formation

Pure conductive polymer hydrogels have many advantages owing to their excellent electrical properties, but they exhibit low stretchability and difficulty in handling without fracturing. Treatment with Triton X-100 to enhance the stretchability of pure conductive polymer hydrogels has been reported, but the stretchability was still insufficient (< 60%) [90]. In contrast, conductive materials with non-conductive polymers, such as poly(vinyl alcohol) (PVA) and polyacrylic acid (PAAc), typically exhibit high stretchability (> 100%) and stable mechanical properties but lack electrical properties.

Recently, various methods have been reported to balance the electrical and mechanical properties of conductive hydrogels. The main objective of these methods is to improve the interconnections between conductive nanocomposites dispersed in a non-conductive polymer hydrogel matrix and to generate percolating conductive nanocomposite networks. This percolating conductive network minimizes the conductive path failure by insulating the domain and increasing the conductivity of the matrix. The first involves the fabrication of a conductive hydrogel from a pure conductive polymer hydrogel (Fig. 2c). Monomers such as acrylic acid can penetrate a pure conductive polymer hydrogel, and an interpenetrated non-conductive network is generated after polymerization. This process affects the conductive polymer network but maintains a percolating conductive network with high conductivity (> 20 S/m) and improved mechanical properties (stretched over 100%) [93, 94]. In other methods, percolating conductive networks are generated from conductive nanocomposites. When using conductive polymers, percolating conductive networks can be generated and improved by the addition of ethylene glycol [95, 96], crosslinkers such as phytic acid, which act as anchoring points for conductive polymers [18, 97, 98], and acid treatment (Fig. 2d) [99]. Such chemical treatment for formation of percolation path in the hydrogel results in volumetric shrinkage of the hydrogels capable of exhibiting a more densely packed structure [99]. That is, this shrinkage can increase the ratio of the conductive domain to the non-conductive domain (e.g., non-conductive polymers, water), which helps achieving high conductivity. In addition, micropatterning strategies have been reported to generate percolating conductive networks using metal nanoparticles, CNT, and GO. First, microgels (hydrogel microparticles) are used to embed conductive nanocomposites on a surface (Fig. 2e) [100, 101]. After the microgels are assembled, a spatially defined percolating conductive network is formed through the microgel surfaces. Therefore, conductive hydrogels fabricated using this method exhibit higher conductivity than normal bulk conductive hydrogels [100]. The second strategy uses spatial rearrangement of conductive nanocomposites through freezing (Fig. 2f) [102]. During the freezing of the aqueous solution, ice crystals are formed, and conductive nanocomposites are located in the region surrounding the ice crystals. A percolating conductive network is formed by this special arrangement and is maintained by crosslinking after removal of the ice crystals [102]. Additionally, the amounts of metal nanoparticles, CNT, and GO are frequently limited during conductive hydrogel fabrication owing to their intrinsic mechanical properties. In contrast, liquid metals such as EGaIn are relatively free from this problem owing to their intrinsic softness and stretchability. Recently, a strategy to assemble liquid metal particles in a hydrogel polymer matrix with ultrahigh conductivity (> 1,000,000 S/m), high stretchability (> 700%), and low modulus (< 200 kPa), even after the addition of 74.4 v/v% liquid metal, was reported [37]. After embedding the liquid-metal particles into the polymer matrix, the acoustic field generates liquid-metal nanoparticles at the particle surfaces. These nanoparticles interconnect with nearby particles to form percolating conductive networks.

## Printing techniques for conductive hydrogel ink

Conventional methods for fabricating conductive hydrogel structures by molding, solvent treatment, and annealing are limited by low resolution, poor interfacial bonding, complex post-processing steps, and environmental hazards. Hence, 3D printing offers a potential solution to overcome these challenges by enabling precise control over the shape, size, and functionality of conductive hydrogel structures. Particularly, considering that one of the most important applications using 3D printing is tissue engineering, the biological tissues have intrinsic function based on coherent cell-to-cell communications within 3D objects geometries. In comparison with 2D structures, such 3D geometries distinctively affect cellular behavior, such as migration, differentiation, and proliferation [103]. Additionally, recent approaches for fabricating 3D bioelectronics to stably stimulate the tissues and record their electrophysiological signals can be deliberately achieved through versatile 3D printing techniques [104]. These 3D printings in bioelectronics field offer freedom of design in 3D space [89] and facile and scalable formation of dense interlayer with electrical connectivity that is crucial for neural electrode performance [105].

**Fig. 3:**
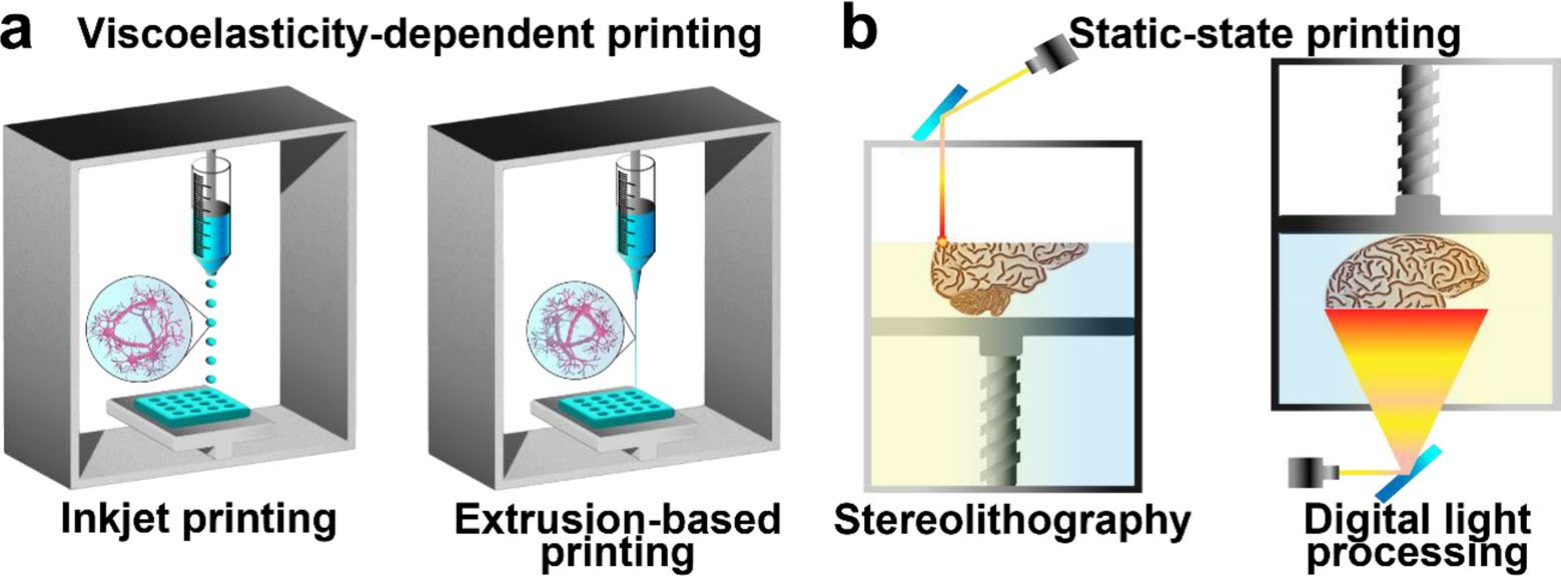
Schematic of 3D printing techniques to fabricate conductive hydrogels. **a** Viscoelastic-dependent printing of conductive hydrogel ink containing conductive particles or conductive polymers (inkjet or extrusion). **b** Static-state printing via photopolymerization of conductive hydrogel ink (DLP or SLA)

Recent advancements in 3D hydrogel-printing technology have enabled the precise fabrication of conductive hydrogels with complex geometries, creating new avenues for a wide range of biomedical applications. To ensure successful printing of the construct, an optimal hydrogel ink that is tailored to a specific printing technique should be prepared. The properties of the hydrogel ink are governed by the type and degree of polymer interactions within the network; therefore, design factors, such as resolution and crosslinking methods, are of utmost importance. For the ink to solidify into the intended architecture, the mechanical and chemical properties of both the precursors and post-print applications should be considered. Therefore, the selection of an appropriate crosslinking method is critical. Current state-of-the-art printing mechanisms can be divided into two categories: viscoelasticity-dependent and static-state printing (Fig. 3). In this section, the various crosslinking mechanisms and printing techniques required to obtain the desired mechanical and chemical properties of the printed constructs are discussed.

### Viscoelasticity-dependent printing

Hydrogels are soft, tissue-like materials with a high-water content, which contributes to their unique viscoelastic characteristics and shear-thinning behavior. Thus, the non-Newtonian trait of a hydrogel, in which the viscosity decreases with increasing shear or stress, enables the movement of the hydrogel through smaller confined spaces such as needles, thereby enabling the hydrogel to be extrudable for use in injection ink. Viscoelasticity-dependent printing techniques involve inkjet printing and extrusion-based printing, which rely on the viscosity of the ink to control the printing process.

#### Inkjet printing

Inkjet printing is a popular method in which small ink droplets are used to create high-resolution structures on various substrates. The inkjet printing of hydrogels depends on the small droplets formed by the pressure pulse at the nozzle. The size and velocity of the droplets are controlled by adjusting the pressure pulse and nozzle size. Inkjet printing has several advantages over other printing techniques, including the ease of hydrogel preparation and suitable viscosity. This reduces the complexity of the printing process and makes it more cost-effective for bioelectric applications, including tissue engineering [106] and biosensing [107–109]. Therefore, these viscoelasticity-dependent inkjet printing can be particularly viable when cells are encapsulated within the ink to be used as a bioink [110]. Owing to the relatively low shear stress on cells during printing, high cell viability and ion functionality are achieved for the bioprinting of neural tissues [111, 112]. Inkjet printing can also be used to fabricate biosensors by depositing biomolecules on conductive substrates with high electrochemical activity [39, 113].

However, inkjet printing also has limitations such as limited printing resolution, the relatively unstable nature of the hydrogel ink, and the necessity for post-processing or for a sacrificial layer. The printing resolution is limited by the size of the droplets, which depends on the viscosity of the hydrogel ink and the nozzle diameter. For a higher resolution, low-viscosity inks and small nozzles are required, but they may compromise the stability and uniformity of the droplets [114]. Therefore, further research is required to optimize the printing parameters and ink formulations to improve the printing resolution, speed, and stability of the printed structures.

#### Extrusion-based printing

Injectable hydrogels are commonly encapsulated in syringes and can be used for diverse biomedical applications. One such application is direct ink writing (DIW). DIW enables in situ writing of ink at a specific site. Jin et al. demonstrated a conductive on-tissue DIW hydrogel ink composed of multiple hydrogen bonds between the cellulose backbone, tannic acid, and a metal-phenol coordinate network between the tannic acid and metal ions [44]. In another study by Jiang et al., which is more closely related to 3D printing, the authors demonstrated the fabrication of a tailored hydrogel structure using a freeze-and-thaw process following the DIW of PVA and carrageenan hydrogel ink [115]. As such, DIW and extrusion-based printing are used interchangeably by researchers [116–119]. However, in most cases, DIW can be used as a preliminary procedure for testing the injectability and facile printability of complex geometries [80, 120–123].

Extrusion-based printing is one of the most widely used printing techniques for fabricating 3D conductive hydrogel structures. Stable and complex printed structures with high resolution can be achieved by controlling the polymerization of the conductive components and optimizing the viscoelasticity of the ink [124, 125]. Recent approaches to the extrusion-based printing of conductive hydrogels utilize self-healing properties to stabilize post-printing structures [126, 127] or tough conductive hydrogels (TCHs) assisted by an in situ photo-crosslinking strategy to reinforce the modulus of the printed structures [128]. The main advantages of extrusion-based printing are its simplicity, versatility, low cost, and potential to upscale structures. However, the resolution of extrusion-based printing is limited to ~100 μm, which largely depends on the inner diameter of the extrusion needle, and constructs with a height require printing in a support bath and sacrificial layers [129]. Furthermore, determining the optimal rheological properties requires a rigorous trial-and-error process. Despite these disadvantages, extrusion-based printing is favored by researchers of conductive hydrogel ink for its potential to create unique and complex structures like hollow tubules, pyramid, ear, and hourglass shapes and its relatively fewer limitations in utilizing the ink [130, 131]. Extrusion-based printing of conductive hydrogels can be applied to fabricate the objects for spinal cord injury [132], artificial epidermis [133, 134], glucose sensor [36], and strain sensor [118, 135–139].

### Static-state 3D printing

Static-state 3D printing technologies are used to create 3D objects by curing materials through vat polymerization (visible light [140] and laser or UV [141–143]). Thus, lithography-based printing of hydrogels is a versatile technique that employs photoreactive moieties in the polymer network to fabricate high-resolution patterns and structures with precise control over the architectural design and even the spatial distribution of the hydrogel ink. Among the various printing techniques that stationarily print polymerized products, stereolithography (SLA) and digital light processing (DLP) are two of the most prominent irradiation techniques used.

#### Stereolithography

SLA uses ultraviolet light to trace the shape of each layer, and the reacted photocurable moieties crosslink to a designated pattern in each layer. SLA techniques have many advantages, such as high accuracy, which can be controlled by the laser spot size and z-axis step increase of each layer [144]. However, brittleness and poor toughness owing to an inhomogeneous polymer architecture and high crosslink density are significant limitations of SLA. In light of these limitations, Keate et al. reported micro-continuous liquid interface production (μCLIP) SLA technique to build myoblast promoting scaffolds [145], and Hui et al. reported on the SLA-based 3D printing of conductive silver–hydrogel circuits embedded within an alginate–polyacrylamide hydrogel matrix as a sacrificial bed [146]. SLA has advantages such as high resolution, high speed, and a wide range of printable materials, allowing its usage in versatile applications, such as human-machine interface [147, 148]. However, a post-printing procedure is essential because of the shrinkage or weakening of the print, which makes it challenging to fabricate highly reproducible samples [149].

#### Digital light processing

DLP is similar to SLA; it is a fast lithography process that projects light over an entire cross-sectional layer simultaneously. In DLP, a thin layer of photocurable resin that lies between the bottom of a printed piece and an optically transparent window is photo-cured. The projector dictates the shape of each level using a digital mirror. After each level is printed, the printed piece is moved up by a thick layer. DLP-based 3D printing can be used to assemble conductive hydrogels for flexible sensors [143, 150, 151] and electrochemical biosensors [152]. Zhu et al. developed a DLP 3D printing method using PEDOT:PSS-PAAm as the conductive ink and poly(2-hydroxyethyl acrylate) as the insulating ink to print electroluminescent devices and capacitive sensors with a strong interface [153]. In another example, Ge et al. developed a highly stretchable hydrogel from a photopolymerized acrylamide– poly(ethylene glycol) diacrylate (PEGDA) DLP construct for use as a strain sensor [154].

A major disadvantage of static-state printing methods is the toxicity caused by the ROS generated during the curing of photoreactive materials, which limits their biomedical applications in vivo. In addition, both SLA and DLP face challenges such as low conductivity, poor interfacial bonding between different materials, limited material selection, low mechanical strength, shrinkage during curing, and toxicity. Therefore, current approaches for static-state 3D printing of conductive hydrogels require further improvements in terms of material design, fabrication process, and device integration.

## 3D printable conductive hydrogel ink properties

**Fig. 4:**
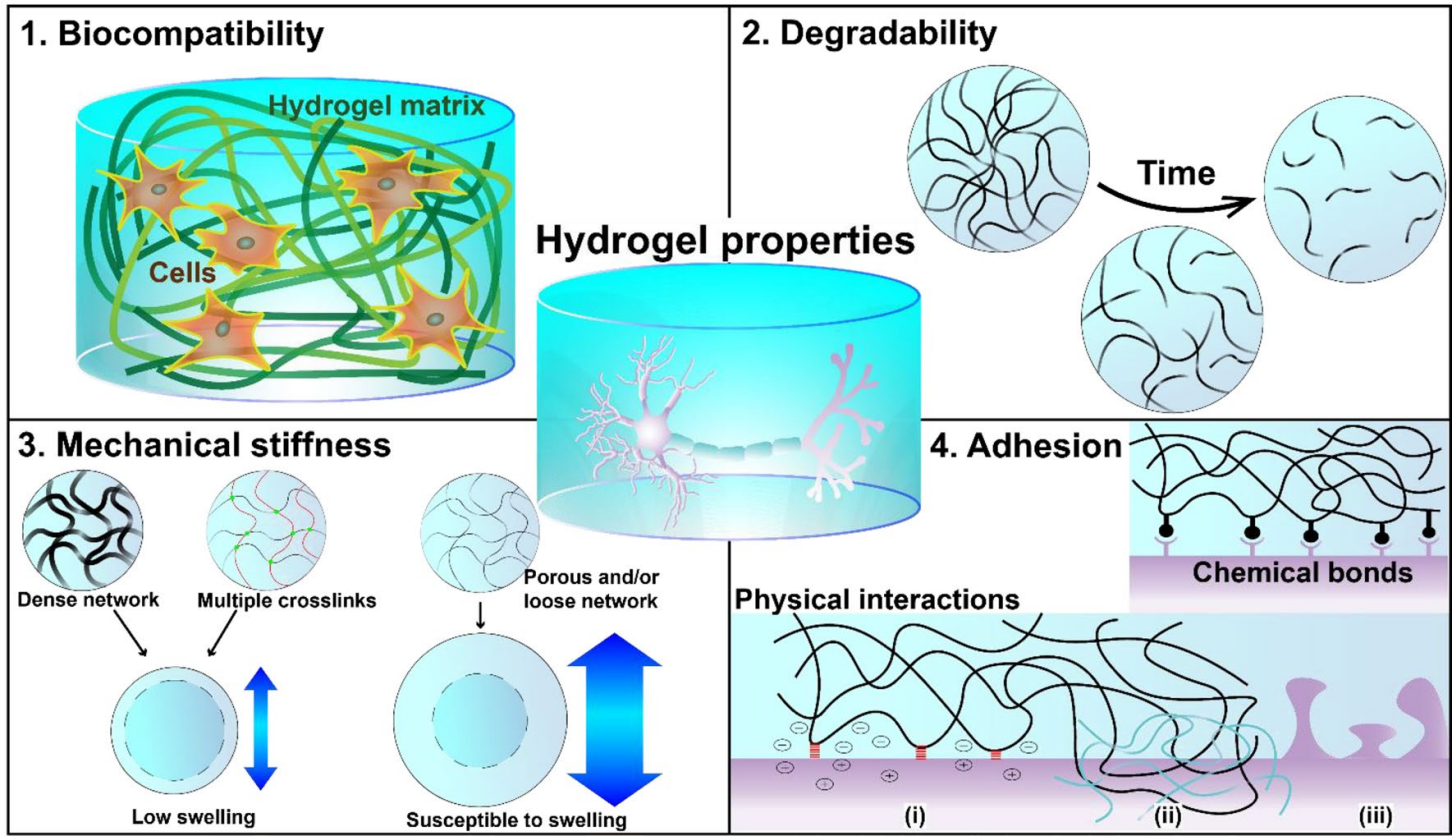
Schematic diagram of different physicochemical properties of conductive hydrogel: (top left) biocompatibility, (top right) degradability of polymer chains, (bottom left) mechanical stiffness of matrix for swelling design, and (bottom right) adhesion from chemical bonds and physical interactions: (i) electrostatic interactions, (ii) polymer chain entanglement, and (iii) mechanical interlocking

In addition to conductivity, applications in neural engineering require biointerfaces with native tissue-like mechanical properties (e.g., degradability, mechanical stiffness, and adhesion) (Fig. 4). However, many current approaches to engineering such a biointerface often face trade-offs between conductivity and mechanical properties. Therefore, the balance between the physical and conductive properties that can provide a biocompatible and stable implant for long-term use should be considered.

### Biocompatibility

Biocompatibility refers to the ability of a material to interact with biological tissues and systems without causing adverse effects such as inflammation, toxicity, or immune rejection. Obtaining minimal or nonexistent immune responses is essential for cell growth or survival and thus for designing neural tissue engineering (Fig. 4). Biocompatibility is influenced by various factors such as material composition, surface mechanical properties, electrical properties, and degradation behavior. Therefore, the biocompatibility of 3D printed conductive hydrogel should be evaluated *in vitro* and in vivo prior to their clinical application.

#### Toxicity of conductive additives

The toxicity of an engineered conductive hydrogel structure can result from aggregates or ROS generated from tissue-material abrasion via mechanical mismatch or from cytotoxic moieties such as aldehydes or metal ions, respectively [155–157]. Therefore, the biocompatibility of conductive additives is a general concern in bioelectronics. Carbon-based additives such as graphene and CNTs are popular conductive hydrogel solutions that increase the conductivity [101, 158, 159]. However, the brittle and sharp surfaces of these additives are known to cause inflammation and oxidative stress in the surrounding cells, limiting their use in tissue engineering applications [66, 160, 161]. Although there have been biocompatible applications of these additives at low concentrations in reduced amounts, percolation problems resulting from low volume decrease the conductivity of the hydrogel [162]. Similarly, metal-based additives, such as Ag and Pt, are also known to cause oxidative stress. Therefore, hydrogels with high volumetric capacitances induced by functionalized or modified additives are a current trend in neural engineering [163]. For example, Deo et al. recently reported a nanoengineered 3D printable hydrogel ink from thiolated gelatin and molybdenum disulfide (MoS_2_) owing to its high biocompatibility, conductivity, and fabrication capability for complex 3D structures [49]. In addition, nanoengineered MXene hydrogels have recently been developed for 3D printing applications [120, 164]; however, only a few reports have discussed their potency in neural tissue engineering [165]. Finally, the soft and flexible mechanical properties of liquid metals have also been investigated for their potential to minimize adverse cytotoxicity [46, 47, 166]. However, their biocompatibility for neural tissue engineering has not yet been confirmed.

Conducting polymers such as PANI [167], PPy [168–170], and PEDOT:PSS [35, 171–173] are used within hydrogels to improve their conductivity while preserving biocompatibility. These organic-based additives chemically interact with polymer chains such that they are homogenously mixed into the conductive ink for 3D printing. Overall, conductive additives that interpenetrate biocompatible polymers remain the most widely used approach in neural tissue engineering applications. For example, hyaluronic acid was reported to inhibit glial scar formation after brain damage, thereby preventing fibrous tissue ingrowth at an injury site [174]. Nevertheless, by ensuring biocompatibility, 3D printed conductive hydrogel offer several advantages for neural engineering in terms of biodegradability, flexibility, porosity, and tunability.

### Degradability

One of the advantages of hydrogel-based implants is their degradability and resorption in the respiratory system. A clinical study on biodegradable and nondegrading conduits found that degradation improved nerve growth and function [175]. Healthy tissue is regenerated at the site of the implant as the hydrogel degrades, providing space for the infiltration of the building blocks. As hydrogel degradation is cleavage of bonds and shortening of polymer chains, it depends on the polymer chain length and concentration, type and degree of crosslinking, and in vivo conditions (Fig. 4). Controlling its degradability is often difficult because of complex physiological functions when applied to the body. Furthermore, for successful tissue remodeling in tissue engineering applications, the neuronal cell proliferation rate must match the hydrogel degradation kinetics. Therefore, the degradation mechanisms of hydrogels must be considered for the specific characteristics of the target tissues [176] or coexist with stable performance [138].

#### Hydrolysis

Hydrophilic polymer chains are prone to hydrolysis, which involves the cleavage of ester or amide bonds due to their affinity to water [148, 177]. Hydrolytic degradation is common in hydrogels in which the polymers are often hydrophilic. Therefore, controlling the hydrophilicity of the overall hydrogel network enables the modulation of the degradation behavior as well as the investigation of other controlled releases of small components [141]. Dutta et al. achieved degradation-controlled osteogenesis within a 3D printed GelMA-PPy-Fe conductive gel by tightly crosslinking the network for nutrient uptake by cells [128].

#### Enzymatic degradation

Enzymatic degradation is specific to hydrogels created from biopolymers such as chitosan, hyaluronic acid, collagen, and gelatin, which react with specific matrix metalloproteinases (MMPs). These enzymes that participate in enzymatic degradation include lysozymes, hyaluronidases, collagenases, and proteases [178–181]. Enzymatic degradation occurs by cleavage of the polymer chains at the target site encoded with a specific enzyme. After degradation, smaller polymer fragments are either safely absorbed by cell or transported through the bloodstream and cleared from body through the renal excretion [182]. However, enzymatic degradation often causes uncontrolled degradation of the hydrogel structure. Deo et al. observed resistance to enzyme degradation in gelatin-containing conductive hydrogels. Overall, they demonstrated that the crosslinking of gelatin hydrogels by MoS_2_ resulted in structural stability under both hydrolytic and enzymatic conditions for a prolonged period [49].

### Mechanical stiffness

The mechanical properties of the hydrogels with similar modulus matching to the tissues are key features for supporting the cells to be encapsulated or surrounded. In 2006, Engler et al. reported that stem cell fates are dictated by mechanical cues [183]. It is important to engineer appropriately the matrix stiffness for maturation of neural stem cells in an aspect of their morphology, extension, and branching [184, 185]. Particularly, the extracellular matrix-like microenvironment provided by hydrogel scaffolds can direct either cell–cell or cell–matrix interactions for the development of desirable neuronal functions [186]. Thus, controlling a hydrogel’s modulus to match the modulus of the surrounding tissue without compromising its electrical and biological properties will improve the signaling and transport of different cytokines and growth factors for neural tissue engineering, particularly in regenerative medicine.

#### Swelling

Typically, hydrogels are susceptible to swelling, and uncontrollable swollen networks can lead to irreversible damage to their structural integrity (Fig. 4). In addition, the increased thickness due to swelling applies pressure to the contacting tissue and impedes blood flow, causing secondary complications. However, controlling the swelling ratio may enhance the degree of drug delivery and cell differentiation by providing a loose network for cytokine and cell infiltration in tissue-engineering applications. Therefore, it is necessary to modulate swelling resistance to humid physiological environments relevant to its function. Swelling resistance is the ability of a hydrogel to resist deformation and volume changes when immersed in an aqueous environment of the human body, and the degree of resistance depends on the physicochemical interactions in the hydrogel network. A common method to increase resistance to swelling is to increase the concentration and crosslinking amount of the hydrogel network to make it dense. Hydrogels with multiple crosslinks via ionic interactions [80, 159, 187], metal-ion coordination bonding [188], or covalent crosslinking [189, 190] provide flexible and tunable mechanical properties that enable the swelling ratio to be adjusted for specific applications.

#### Porosity

The porosity of a hydrogel is related to its permeability across the hydrogel. Small pore sizes reduce permeability and recovery is slowed, whereas large pore sizes can lead to instability and failure, and small nutrients, such as oxygen and growth factors, must be able to infiltrate deep within the hydrogel network to promote homogeneous functions and regeneration [171]. Distler et al. reported on the fabrication of a 3D open-porous electrically conductive scaffold for tissue engineering and observed that the porous structure increased the cell-seeding efficiency along with electrical cell stimulation functionality [126].

From an engineering perspective, the porosity can influence the mechanical stiffness of conductive hydrogels in two ways: by affecting the stress distribution and by providing channels for fluid flow. On one hand, porosity can reduce the mechanical stiffness of conductive hydrogels by creating defects and cracks in the network, which can lower the stress-bearing capacity and fractures. On the other hand, porosity can increase the mechanical stiffness of conductive hydrogels by facilitating fluid drainage and relieving internal pressure, which can prevent swelling and enhance elasticity. Therefore, porosity should be optimized to balance these effects and achieve the desired mechanical stiffness for different applications.

### Adhesion

For stable electrical flow between a conductive hydrogel and biological tissue, strong and stable adhesion is essential for the accurate transfer of electrical stimulation and recording at the tissue–scaffold interface. In this section, we discuss the physical and chemical adhesion mechanisms that are considered when functionalizing conducting hydrogels with adhesive properties (Fig. 4).

#### Physical interactions

Physical interactions refer to non-covalent forces that mediate the adhesion between conductive hydrogels and substrates such as tissues. These intermolecular interactions include van der Waals forces [191], electrostatic forces [192], hydrogen bonding [36, 136, 139, 192, 193], and mechanical interlocking [194] that contribute to adhesion (e.g., polymer chain entanglement on tissue surface) and self-healing properties of hydrogel. These forces depend on the surface properties of both the hydrogel and tissue, such as roughness, charge, polarity, and porosity. Conformal physical interactions result in mechanical modulus matching. For example, rougher surfaces can increase the contact area and interlocking between the hydrogel and tissue, thereby enhancing adhesion strength and conformability [194]. Similarly, charged surfaces can induce electrostatic attraction or repulsion at the interface depending on their relative polarity [157, 195].

#### Chemical bonds

Chemical interactions refer to the covalent bonds that form between conductive hydrogels and tissue owing to biological factors or external chemical precursors, and these covalent bonds include cis-diol bonds, ester bonds, amide bonds, disulfide bonds, and click chemistry [134, 142, 193, 196–198]. These bonds are more specific but provide stronger adhesion energy than previously mentioned physical interactions. For example, conductive hydrogels containing functional groups (e.g., catechol or aldehydes) which can form covalent bonds with the tissues, show strong adhesiveness. Catechol groups inspired by marine mussels’ adhesion and tannic acid derived by plant extracts react with amino acid residues present in the tissues (e.g., lysine and cysteine) via Michael addition or Schiff’s base formation [44, 73, 83, 199]. Also, aldehyde-containing hydrogels can form imine bonds with amino groups on tissue surface through Schiff’s base formation [47, 199].

## Application

### Neural tissue engineering

**Fig. 5:**
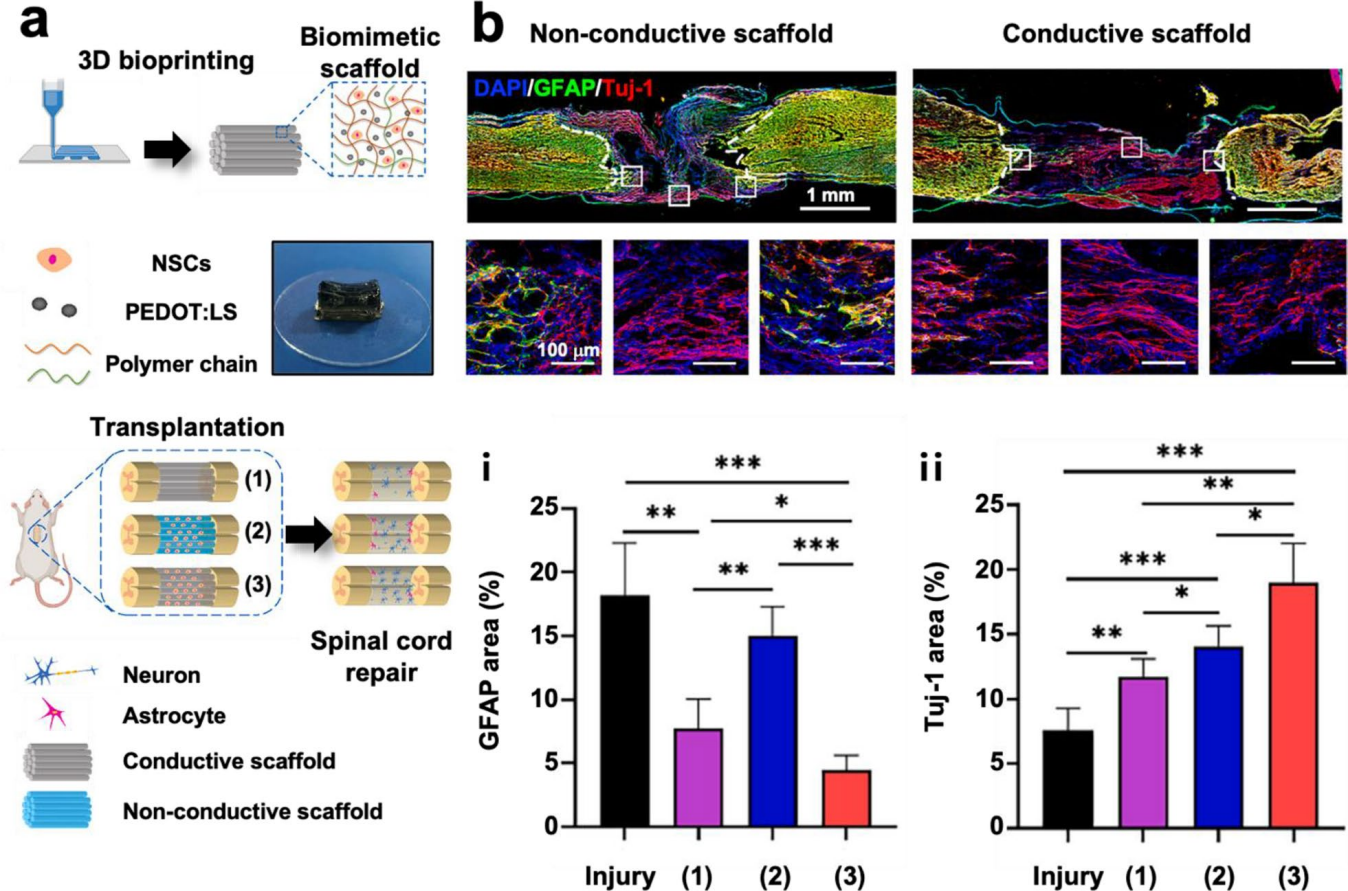
Neural tissue engineering applications of 3D printable conductive hydrogel ink. **a** Illustration of 3D printed biomimetic scaffolds using sulfonated lignin (LS)-doped PEDOT containing conductive hydrogel ink and transplantation to the spinal cord injury model. **b** Immunofluorescence staining exhibit that the 3D printed biomimetic scaffolds (i) inhibit the formation of glial scar and (ii) promote neuronal regeneration. Reproduced with permission from [215], copyright Elsevier, 2023

In the process of neuronal cell growth and differentiations, endogenous bioelectrical signaling has been known as a crucial factor to enhance the patterning and formation of the synapse [200, 201]. Conductive hydrogels, as a scaffold, mimic the electrophysiological environment of the neuronal tissues and help improve bioelectrical signal propagation between cells [15]. For example, conductive hydrogels composed of PPy, PEDOT:PSS, and CNT increase cell adhesion and proliferation [73, 79, 95, 202, 203] and promote neuronal differentiation of stem cells [102, 169, 203–206]. Tringides et al. demonstrated that conductive viscoelastic alginate hydrogels with CNT and graphene trigger greater differentiation from neural progenitor cells (NPCs) to neuron, astrocytes, or myelinating oligodendrocytes than those in non-conductive hydrogels [102]. Also, when the conductive hydrogels are in vivo implanted to injured sites of the neuronal tissues, neural tissue regeneration is improved [11, 40, 48, 54, 58, 195, 207, 208]. Zhou et al. developed tannic acid-doped PPy hydrogels for enhancing spinal cord repair [11]. Locomotor activity of mice was evaluated 6 weeks after spinal cord injury, and conductive hydrogel scaffold implantation enabled faster and better restoration of locomotor activity. Recently, the significance of biomimetic structures for the regeneration of neural tissues such as the spinal cord and sciatic nerves has emerged. The implementation of an anisotropic structure and heterogeneity of the white and gray matter of the spinal cord promotes regeneration in the spinal cord injury model [209, 210] and nerve guide conduits with porous, multichannel structures exhibit effective peripheral nerve regeneration [211–214]. Therefore, interest in 3D printable conductive hydrogel inks has increased to achieve the synergistic effect of conductive hydrogels and biomimetic structures for neural tissue engineering. For example, Gao et al. printed a PEDOT-containing conductive hydrogel ink with neural stem cells (NSCs) into a parallel linear pattern to mimic the anisotropic structure of the spinal cord and implanted it into a rat spinal cord injury model (Fig. 5a) [215]. A 3D printed conductive hydrogel scaffold promoted NSC differentiation into myelinated neuron and exhibited a more effective spinal cord regeneration than previous result from their research group using non-conductive hydrogel scaffold (Fig. 5b) [216]. Serafin et al. applied a 3D printable conductive hydrogel to a spinal cord injury model, which exhibited superior axonal growth [171]. 3D printable conductive hydrogels are also useful for cell-patterned in vitro models. For example, Kuzmenko et al. printed conductive hydrogels on non-conductive hydrogels and showed that neuroblastoma cells prefer to grow on conductive hydrogel surfaces [202].

### Bioelectronics

**Fig. 6:**
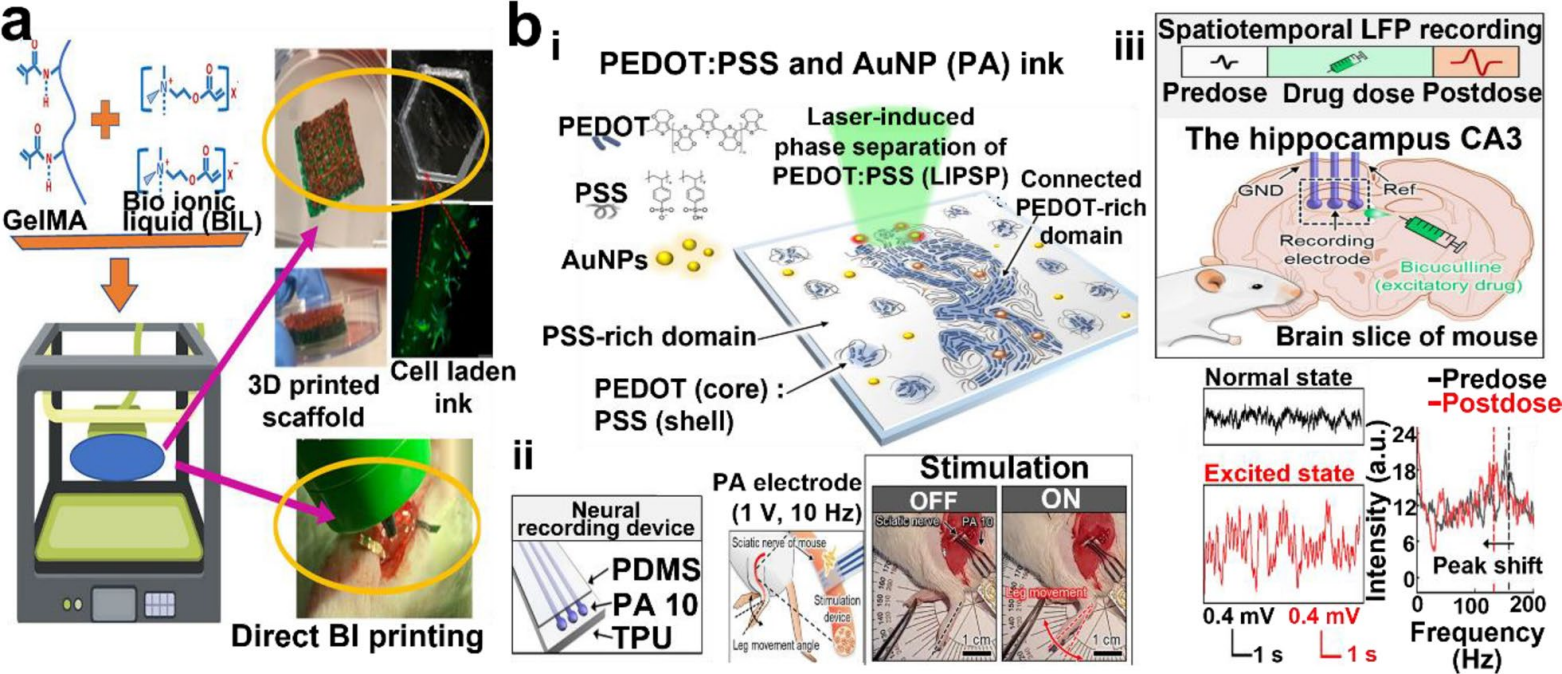
Bioelectronic application of conductive hydrogel. **a** 3D printing conductive ink on muscle and in vivo actuation of electrical stimulation. Reproduced with permission from [219], copyright Elsevier, 2023. **b** Digital light patterning of PA (PEDOT:PSS/AuNP) hydrogel for neural signal recording and stimulation: i) schematic of selective patterning of ink for fabrication of neural recording device, ii) neural stimulation with PA electrode insulated with PDMS (polydimethylsiloxane) and TPU (thermoplastic polyurethane) to induce leg movement, and iii) LFP (local field potential) recording on mouse brain slice and peak shift observed for excited state (bicuculline drug dose). Reproduced with permission from [35], copyright American Association for the Advancement of Science, 2022

Conductive hydrogels have emerged as promising candidates for bioelectronics with potential applications in neural engineering. However, significant challenges remain for the manufacturing of hydrogel-based devices owing to their complicated mold-casting process. Although conductive hydrogels offer the advantage of being implantable in vivo without tissue damage, challenges remain in directly writing complex circuits owing to their low tissue adhesion and heterogeneous mechanical properties. Therefore, in recent years, printable conductive hydrogels have been developed to combine the advantages of hydrogels and conductive polymers for bioelectronic applications. These hydrogels can be fabricated using various printing techniques, as discussed previously, into flexible and biocompatible devices that can be used in neural engineering applications, such as nerve regeneration using biochemical and bioelectrical delivery from 3D co-axial printed chips for proliferation and differentiation (Fig. 6a) [217] or 3D printed skin-like stress/pressure sensors [218].

Similarly, 3D printed conductive hydrogels have been shown to provide electrical and mechanical cues to modulate neural activity and growth, as well as to record neural signals with high sensitivity and resolution. For example, Krishnadoss et al. fabricated a 3D printed multilayer scaffold from choline/GelMA hydrogels, which exhibited bio-interfacing feasibility for bioelectronics in tissue injury [219]. Tissue repair is a key research area in bioelectronics because electrical stimulation to facilitate neuron growth [220] or stem cell differentiation [196] is most compatible with conductive hydrogels. A silver nanowire and methacrylate alginate-based electrical hydrogel patch (ePatch) by Wang et al. exhibited rapid wound closure owing to the migration of proliferative cells in response to electrical stimulation [221].

Another key research area in bioelectronics is the recording of electrophysiological signals. A bioelectrode is a device that enables the transfer of electrical charge between an electrode and an electrolyte, such as a biological tissue or fluid. Bioelectrodes are widely used in electrophysiological studies to measure the electrical activity of cells, tissues, and organs. One of the main challenges of bioelectrodes is overcoming the double-layer capacitance formed at the interface between the electrode and tissue. However, because of the water-rich and polymeric nature of hydrogels, 3D printed bioelectrodes with conductive hydrogels generate volumetric capacitance compared with conventional metallic electrodes with areal capacitance, which ultimately decreases the interfacial impedance, thereby enhancing the performance of the bioelectrode at the interface [9].

In addition to their high conductive performance, hydrogel electrodes have advantages in terms of biocompatibility, flexibility, and adhesion to tissues, enabling high sensitivity to various electrophysiological signals, such as electromyograms (EMGs) and electrocardiograms (ECGs) [222]. For example, a DLP-printed PEDOT:PSS hydrogel was recently used for EMG and ECG recordings [96]. Similarly, PVA/borax/PEDOT:PSS hydrogel electrodes were used as epidermal patches for ECG and EMG monitoring [223] and extrusion printed PEDOT:PSS/rGO electrode arrays were used for EMG and ECG measurements [224]. In another study, triple-network crosslinked hydrogel electrodes composed of PVA, sodium alginate, and PAAm were used for EMG, ECG, respiration, and joint monitoring [166].

**Fig. 7:**
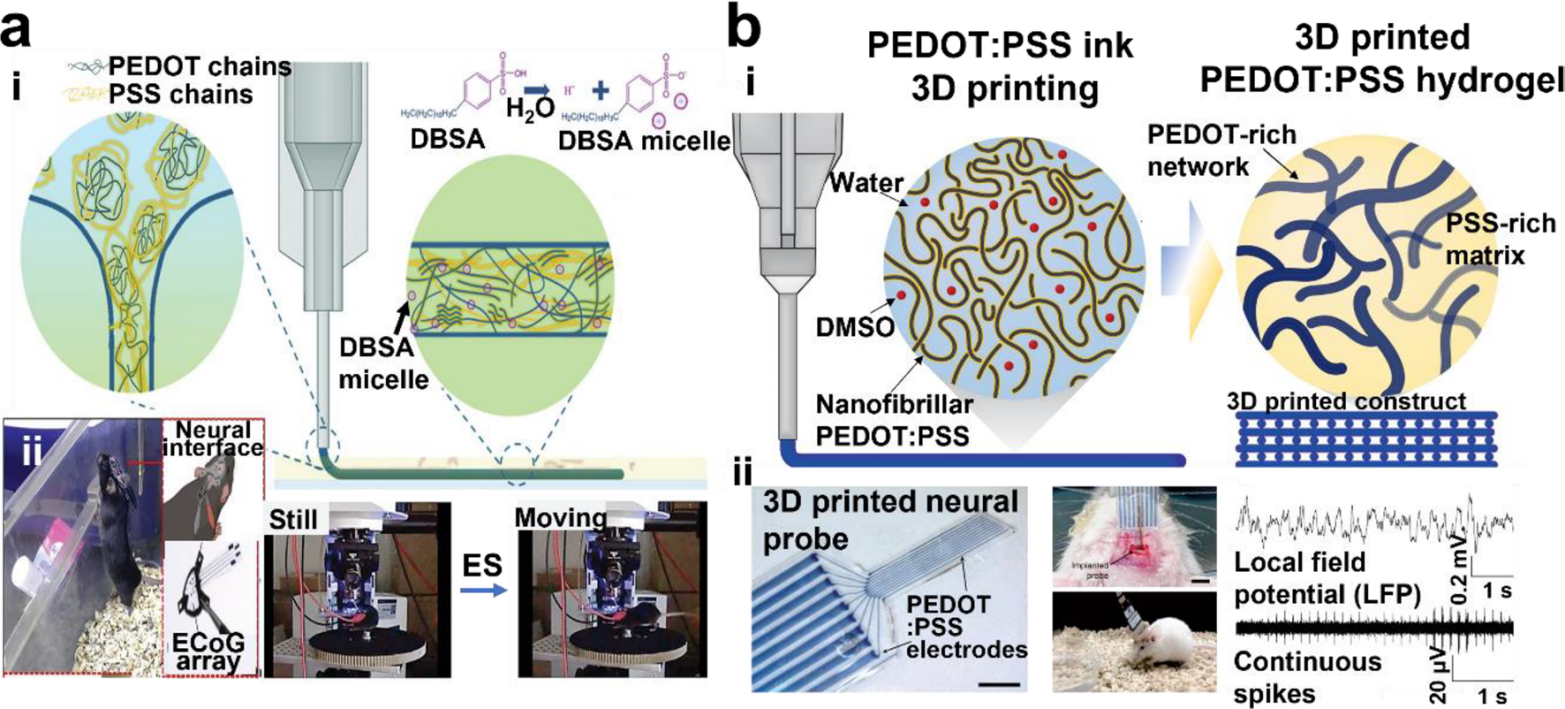
ECoG application of conductive hydrogel. **a** extrusion-based printing of PEDOT:PSS and DBSA micelle ink: (i) ink materials and printing scheme and (ii) printed ECoG array for recording motor functions. Reproduced with permission from [92], copyright John Wiley and Sons, 2022. **b** Purely PEDOT:PSS ink 3D printing: (i) Schematic diagram of PEDOT:PSS network within hydrogel and (ii) ECoG recording from printed neural probe. Reproduced with permission from [89], copyright Springer Nature, 2020

In addition to EMG and ECG signals, other electrophysiological signals such as electrocorticograms (ECoGs), electrooculograms (EOGs), and electroencephalograms (EEGs) can be recorded using 3D printed conductive hydrogels. For example, Won et al. reported a water-stable, digital light patterned PEDOT:PSS hydrogel neural electrode, which can be used for neural signal recording and stimulation over 6 months (Fig. 6b) [35]. In another work, PEDOT:PSS crosslinked with PEGDA and alginate has been used to fabricate self-adhesive and conductive hydrogel electrodes for EOG recording [225]. Additionally, μSLA-printed hydrogel strain sensors were used for recording EOG and EEG signals [226]. In addition to previously mentioned monitoring, real-time recording or stimulation is possible with conductive hydrogel. Long-term stable ECoG array developed separately by Zheng et al. and Yuk et al. successfully recorded bioelectronic response from brain (Fig. 7) [89, 92, 227].

Purely conductive hydrogel-based electrodes may have limitations in bioelectronics owing to their uncontrollable degradability, swelling, and lack of flexibility. Therefore, several novel regeneration interfaces that combine other classes of materials with hydrogels have been developed. For example, Cao et al. reported diabetic wound healing using a combination of electrospun polyurethane nanofibrous membranes and a 3D extrusion-printed choline/GelMA hydrogel [228]. Chronic wound healing was observed owing to the regulated release of doxorubicin from the fabricated conductive wound dressing. In a nerve application study, Hiendlmeier et al. demonstrated peripheral nerve stimulation via a SLA-fabricated 4D cuff electrode that had a bilayer of flexible substrate and hydrogel [229]. In the field of bone regeneration, Li et al. 3D printed a bioactive prosthetic interface with a titanium alloy and a conductive supramolecular hydrogel composed of PVA, chitosan, agarose, and silver nanowires to improve osteoporosis [230]. Therefore, for long-term applications and biocompatibility, conductive hydrogels may be combined with other materials to gain advantages such as the malleability of conductive hydrogels into 3D printing and the lasting structural conformability of elastomers or ceramics. In conclusion, either in pure hydrogel or in combination approaches, printable conductive hydrogels are promising materials for developing advanced neural interfaces and therapies.

## Outlook

**Fig. 8:**
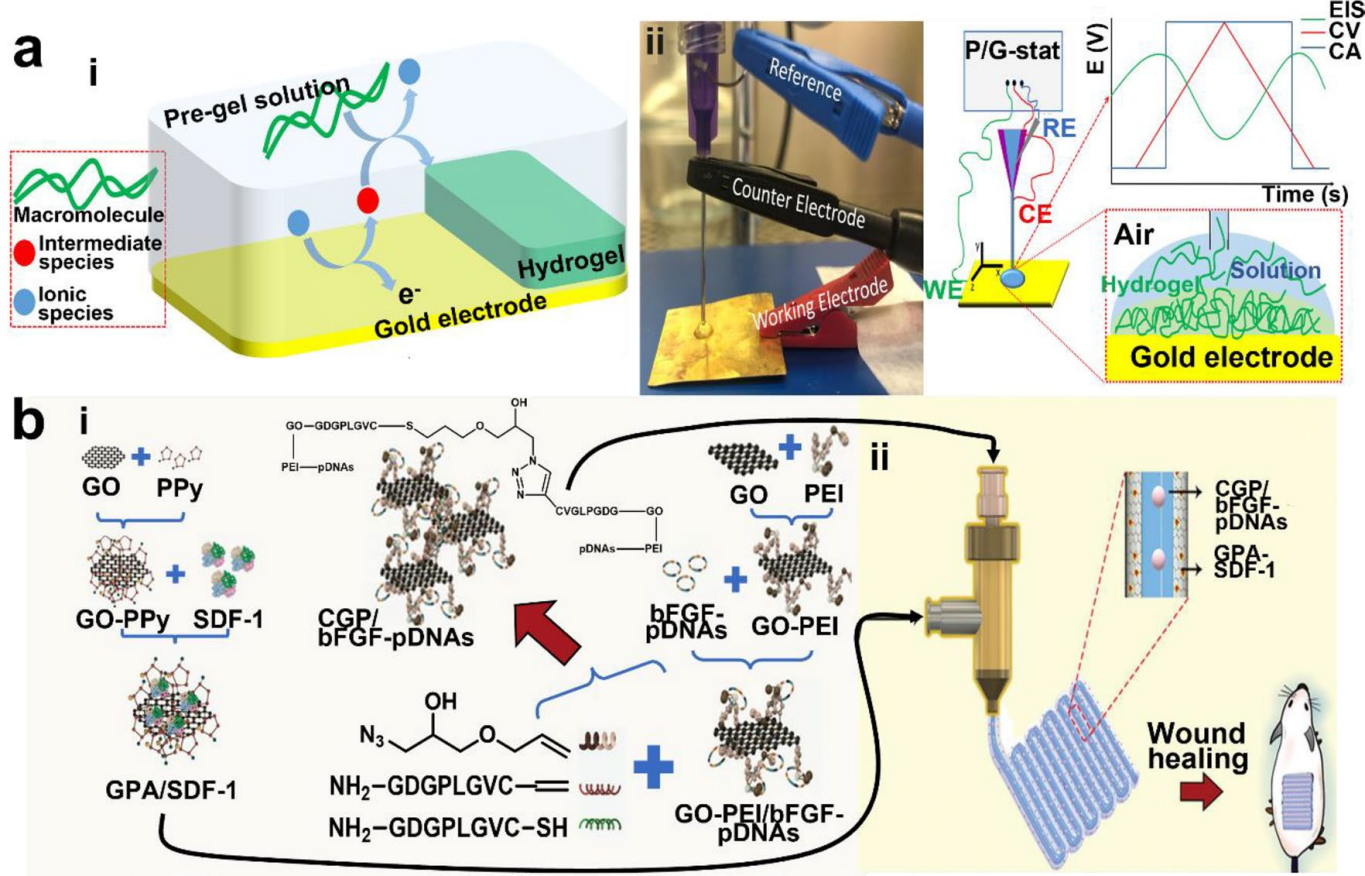
Unconventional printing techniques for conductive hydrogels. **a** Electro-assisted printing via controlled electrochemical reactions: (i) printing mechanism and (ii) printing set-up with P/G-stat (potentiostat/galvanostat). Reproduced with permission from [232], copyright Springer Nature, 2022. **b** 3D co-axial printed chip for skin nerve regeneration in wound: (i) ink materials and preparation, (ii) co-axial printing of core–shell microfiber. Reproduced with permission from [217], copyright John Wiley and Sons, 2020

In conductive hydrogel printing for versatile neural engineering, it is generally understood that the quality of 3D printed structures largely depends on the chemical composition of the hydrogel. Therefore, while many studies have focused on materials associated with 3D printable structures, studies related to selection of optimal printing strategies have not been considered as significant. A few novel engineering methods for printing the structures of conductive hydrogels include combined strategies or augmentation of conventional methods. Orthogonal photochemistry-assisted printing (OPAP), developed by Wei et al., combines 3D extrusion printing with visible-light photogelation to print TCHs that can be used for hydrogel arrays [189]. Ahn et al. utilized air-pressure-assisted pen-nib printing for novel approach to printing electronics [231]. Silva et al. introduced a unique electro-assisted hydrogel deposition of PEDOT and alginate droplets based on electrochemical reactions (Fig. 8a) [232]. Peng et al. demonstrated the co-axial 3D printing of conductive and degradable GO-PPy-alginate chips for skin neuronal differentiation. The shell ink (GPA-SDF-1) was fabricated by anchoring SDF-1 chemokine to GO-PPy in alginate ink while the core ink (CGP/bFGF-pDNAs) was made by conjugating GO with PEI (polyetherimide), transfected with bFGF-pDNA (plasmid DNA), and crosslinked with MMP-2 (matrix metalloproteinase-2) sensitive peptide sequences. The core–shell structure from co-axial printing was stimulated with bioelectrical signal and showed differentiation and maturation of MSCs (mesenchymal stem cells) to functional neuronal cells (Fig. 8b) [217]. Beyond unconventional printing techniques described above, the conductive materials to be loaded in the hydrogel ink can be newly designed for further neural engineering. Using laser irradiation, graphitization of various organic materials (e.g., polydopamine and lignin) and thermal annealing of PEDOT:PSS can be induced [35, 233, 234]. Furthermore, photothermal energy caused by laser irradiation can generate selective conductive path within the hydrogel inks, realizing delicate 3D printing of bioelectronic devices [35, 234]. Regarding these, Miyakoshi et al. developed a hydrogel capacitor through laser-induced lignin graphitization in agarose hydrogel, and Won et al. reported PEDOT:PSS patterning based on laser induced photothermal energy from gold nanoparticles [35, 234]. To sum up, the 3D printing of conductive hydrogels has recently led to remarkable advances in the fabrication of complex and functional structures. Novel 3D printing strategies can solve the limitations of conventional methods such as printing resolution, biocompatibility, and functionality. These printing strategies have the potential to revolutionize and ultimately contribute to the development of a toolbox for designing conductive hydrogels for bioelectronics as well as advanced tissue engineering applications. In the near future, such 3D printed conductive hydrogels would be promising as a tool to electrically stimulate on neural tissues, record their electrophysiological signals with high signal-to-noise ratio and provide neural tissue-mimetic scaffold with improved therapeutic efficacy. Toward clinical translation, the 3D-printed conductive objects, or the conductive hydrogel inks by themselves might allow future close-looped neuroprosthesis capable of stimulation, recording, and restoration of the electrophysiological signals from human body stimulate and can be utilized as therapeutic platform for spinal cord and peripheral nerve.

## Data Availability

The review is based on the published data and sources of data upon which conclusions have been drawn can be found in the reference list.

## References

[1] Sousa JPM, Stratakis E, Mano J, Marques P (2023). Biomater. Adv..

[2] Liu K, Yan L, Li R, Song Z, Ding J, Liu B, Chen X (2022). Adv. Sci..

[3] Gilron R, Little S, Perrone R, Wilt R, de Hemptinne C, Yaroshinsky MS, Racine CA, Wang SS, Ostrem JL, Larson PS, Wang DD, Galifianakis NB, Bledsoe IO, San Luciano M, Dawes HE, Worrell GA, Kremen V, Borton DA, Denison T, Starr PA (2021). Nat. Biotechnol..

[4] Koo J, MacEwan MR, Kang SK, Won SM, Stephen M, Gamble P, Xie Z, Yan Y, Chen YY, Shin J, Birenbaum N, Chung S, Kim SB, Khalifeh J, Harburg DV, Bean K, Paskett M, Kim J, Zohny ZS, Lee SM, Zhang R, Luo K, Ji B, Banks A, Lee HM, Huang Y, Ray WZ, Rogers JA (2018). Nat. Med..

[5] Choi YS, Hsueh YY, Koo J, Yang Q, Avila R, Hu B, Xie Z, Lee G, Ning Z, Liu C, Xu Y, Lee YJ, Zhao W, Fang J, Deng Y, Lee SM, Vazquez-Guardado A, Stepien I, Yan Y, Song JW, Haney C, Oh YS, Liu W, Yoon HJ, Banks A, MacEwan MR, Ameer GA, Ray WZ, Huang Y, Xie T, Franz CK, Li S, Rogers JA (2020). Nat. Commun..

[6] Sanjuan-Alberte P, Whitehead C, Jones JN, Silva JC, Carter N, Kellaway S, Hague RJM, Cabral JMS, Ferreira FC, White LJ, Rawson FJ (2022). iScience..

[7] Barrejon M, Rauti R, Ballerini L, Prato M (2019). ACS Nano.

[8] Green R, Abidian MR (2015). Adv. Mater..

[9] Yuk H, Lu B, Zhao X (2019). Chem. Soc. Rev..

[10] Sunwoo SH, Han SI, Joo H, Cha GD, Kim D, Choi SH, Hyeon T, Kim DH (2020). Matter.

[11] Zhou L, Fan L, Yi X, Zhou ZN, Liu C, Fu RM, Dai C, Wang ZG, Chen XX, Yu P, Chen DF, Tan GX, Wang QY, Ning CY (2018). ACS Nano.

[12] Saha K, Keung AJ, Irwin EF, Li Y, Little L, Schaffer DV, Healy KE (2008). Biophys. J..

[13] Zhang C, Tan Y, Feng JT, Huang C, Liu BY, Fan Z, Xu B, Lu T (2020). ACS Omega.

[14] Wang ZL, Song HY, Chen L, Li WH, Yang DS, Cheng P, Duan HG, Appl ACS (2022). Electron. Mater..

[15] Dvir T, Timko BP, Brigham MD, Naik SR, Karajanagi SS, Levy O, Jin H, Parker KK, Langer R, Kohane DS (2011). Nat. Nanotechnol..

[16] Feig VR, Santhanam S, McConnell KW, Liu K, Azadian M, Brunel LG, Huang Z, Tran H, George PM, Bao Z (2021). Adv. Mater. Technol..

[17] Hsu RS, Li SJ, Fang JH, Lee IC, Chu LA, Lo YC, Lu YJ, Chen YY, Hu SH (2022). Nat. Commun..

[18] An YH, Lee J, Son DU, Kang DH, Park MJ, Cho KW, Kim S, Kim SH, Ko J, Jang MH, Lee JY, Kim DH, Hwang NS (2020). ACS Nano.

[19] Xu LL, Yang Y, Mao YK, Li Z (2022). Adv. Mater. Technol..

[20] Liang QD, Shen ZZ, Sun XG, Yu DH, Liu KW, Mugo SM, Chen W, Wang D, Zhang Q (2023). Adv. Mater..

[21] Hong YJ, Jeong H, Cho KW, Lu N, Kim DH (2019). Adv. Funct. Mater..

[22] Lee M, Rizzo R, Surman F, Zenobi-Wong M (2020). Chem. Rev..

[23] Wang C, Huang W, Zhou Y, He L, He Z, Chen Z, He X, Tian S, Liao J, Lu B, Wei Y, Wang M (2020). Bioact. Mater..

[24] Lind JU, Busbee TA, Valentine AD, Pasqualini FS, Yuan H, Yadid M, Park SJ, Kotikian A, Nesmith AP, Campbell PH, Vlassak JJ, Lewis JA, Parker KK (2017). Nat. Mater..

[25] Min K, Kong JS, Kim J, Kim J, Gao G, Cho DW, Han HH, Appl ACS (2022). Bio Mater..

[26] Sodupe Ortega E, Sanz-Garcia A, Pernia-Espinoza A, Escobedo-Lucea C (2019). Materials.

[27] Li K, Wang D, Zhao K, Song K, Liang J (2020). Talanta.

[28] Zhang X, Huang H, Lang X, Chen Z, Zeng H, Chang Y, Nie Y (2023). Int. J. Biol. Macromol..

[29] Hao Y, Cao B, Deng L, Li J, Ran Z, Wu J, Pang B, Tan J, Luo D, Wu W (2023). Int. J. Bioprint..

[30] Chen Y, Zhang J, Liu X, Wang S, Tao J, Huang Y, Wu W, Li Y, Zhou K, Wei X, Chen S, Li X, Xu X, Cardon L, Qian Z, Gou M (2020). Sci. Adv..

[31] Choi YJ, Jun YJ, Kim DY, Yi HG, Chae SH, Kang J, Lee J, Gao G, Kong JS, Jang J, Chung WK, Rhie JW, Cho DW (2019). Biomaterials.

[32] Antich C, de Vicente J, Jimenez G, Chocarro C, Carrillo E, Montanez E, Galvez-Martin P, Marchal JA (2020). Acta Biomater..

[33] Zhu YZ, Joralmon D, Shan WT, Chen YY, Rong JH, Zhao HY, Xiao SQ, Li XJ (2021). Bio. Des. Manuf..

[34] Gao G, Huang Y, Schilling AF, Hubbell K, Cui X (2018). Adv. Healthc. Mater..

[35] Won D, Kim J, Choi J, Kim H, Han S, Ha I, Bang J, Kim KK, Lee Y, Kim TS, Park JH, Kim CY, Ko SH (2022). Sci. Adv..

[36] Shang Y, Wu C, Hang C, Lu H, Wang Q (2020). Adv. Mater..

[37] Lee W, Kim H, Kang I, Park H, Jung J, Lee H, Park H, Park JS, Yuk JM, Ryu S, Jeong JW, Kang J (2022). Science.

[38] Hsiao LY, Jing L, Li KR, Yang HT, Li Y, Chen PY (2020). Carbon.

[39] Li L, Pan L, Ma Z, Yan K, Cheng W, Shi Y, Yu G (2018). Nano Lett..

[40] J. Xu, C-H. Tai, T-Y. Chen, S-h. Hsu, Chem. Eng. J. **446**, 137180 (2022)

[41] Zhu K, Shin SR, van Kempen T, Li YC, Ponraj V, Nasajpour A, Mandla S, Hu N, Liu X, Leijten J, Lin YD, Hussain MA, Zhang YS, Tamayol A, Khademhosseini A (2017). Adv. Funct. Mater..

[42] Navaei A, Saini H, Christenson W, Sullivan RT, Ros R, Nikkhah M (2016). Acta Biomater..

[43] Wang C, Flynn NT, Langer R (2004). Adv. Mater..

[44] Jin S, Kim Y, Son D, Shin M (2022). Gels.

[45] Baei P, Jalili-Firoozinezhad S, Rajabi-Zeleti S, Tafazzoli-Shadpour M, Baharvand H, Aghdami N (2016). Mater. Sci. Eng. C. Mater. Biol. Appl..

[46] Liao M, Liao H, Ye J, Wan P, Zhang L, Appl ACS (2019). Mater. Inter..

[47] Xu Y, Rothe R, Voigt D, Hauser S, Cui M, Miyagawa T (2021). M Patino Gaillez, T Kurth, M Bornhauser, J Pietzsch, Y Zhang. Nat. Commun..

[48] Yu QN, Jin SC, Wang SC, Xiao HN, Zhao YT (2023). Chem. Eng. J..

[49] Deo KA, Jaiswal MK, Abasi S, Lokhande G, Bhunia S, Nguyen TU, Namkoong M, Darvesh K, Guiseppi-Elie A, Tian L, Gaharwar AK (2022). ACS Nano.

[50] X.J. Zhang, Y.C. Zhang, W.L. Zhang, Y. Dai, F. Xia, Chem. Eng. J. **420**, 130447 (2021)

[51] P.G. Jamkhande, N.W. Ghule, A.H. Bamer, M.G. Kalaskar, J. Drug Deliv. Sci. Tech. **53**, 101174 (2019)

[52] Shin SR, Migliori B, Miccoli B, Li YC, Mostafalu P, Seo J, Mandla S, Enrico A, Antona S, Sabarish R, Zheng T, Pirrami L, Zhang K, Zhang YS, Wan KT, Demarchi D, Dokmeci MR, Khademhosseini A (2018). Adv. Mater..

[53] Zhang W, Xu L, Zhao M, Ma Y, Zheng T, Shi L (2022). Soft Matter.

[54] Park J, Jeon J, Kim B, Lee MS, Park S, Lim J, Yi J, Lee H, Yang HS, Lee JY (2020). Adv. Funct. Mater..

[55] Li Y, He J, Zhou J, Li Z, Liu L, Hu S, Guo B, Wang W (2022). Biomater. Sci..

[56] Choe G, Oh S, Seok JM, Park SA, Lee JY (2019). Nanoscale.

[57] Jo H, Sim M, Kim S, Yang S, Yoo Y, Park JH, Yoon TH, Kim MG, Lee JY (2017). Acta Biomater..

[58] M. Wang, C.G. Wang, M. Chen, M. Luo, Q.X. Chen, B. Lei, Chem. Eng. J. **439**, 135629 (2022)

[59] Han L, Liu KZ, Wang MH, Wang KF, Fang LM, Chen HT, Zhou J, Lu X (2018). Adv. Funct. Mater..

[60] Ryu S, Chou JB, Lee K, Lee D, Hong SH, Zhao R, Lee H, Kim SG (2015). Adv. Mater..

[61] Su GH, Cao J, Zhang XQ, Zhang YL, Yin SY, Jia LY, Guo QQ, Zhang XX, Zhang JH, Zhou T (2020). J. Mater. Chem. A.

[62] Li W, Tao LQ, Kang MC, Li CH, Luo CY, He G, Sang TY, Wang P (2022). Carbohyd. Polym..

[63] Lee J, Manoharan V, Cheung L, Lee S, Cha BH, Newman P, Farzad R, Mehrotra S, Zhang K, Khan F, Ghaderi M, Lin YD, Aftab S, Mostafalu P, Miscuglio M, Li J, Mandal BB, Hussain MA, Wan KT, Tang XS, Khademhosseini A, Shin SR (2019). ACS Nano.

[64] Han S, Wu Q, Zhu J, Zhang J, Chen A, Su S, Liu J, Huang J, Yang X, Guan L (2023). Mater. Horiz..

[65] Wang YL, Han L, Zhang XL, Cao L, Hu K, Li LH, Wei Y (2022). J. Tissue Eng. Regen. M.

[66] Zhang Y, Ali SF, Dervishi E, Xu Y, Li Z, Casciano D, Biris AS (2010). ACS Nano.

[67] Schrand AM, Dai L, Schlager JJ, Hussain SM, Osawa E (2007). Diam. Relat. Mater..

[68] Sun XM, Liu Z, Welsher K, Robinson JT, Goodwin A, Zaric S, Dai HJ (2008). Nano Res..

[69] Hong GS, Lee JC, Robinson JT, Raaz U, Xie LM, Huang NF, Cooke JP, Dai HJ (2012). Nat. Med..

[70] Chong Y, Ma YF, Shen H, Tu XL, Zhou X, Xu JY, Dai JW, Fan SJ, Zhang ZJ (2014). Biomaterials.

[71] Poland CA, Duffin R, Kinloch I, Maynard A, Wallace WAH, Seaton A, Stone V, Brown S, MacNee W, Donaldson K (2008). Nat. Nanotech..

[72] Liang S, Zhang Y, Wang H, Xu Z, Chen J, Bao R, Tan B, Cui Y, Fan G, Wang W, Wang W, Liu W (2018). Adv. Mater..

[73] Yang S, Jang L, Kim S, Yang J, Yang K, Cho SW, Lee JY (2016). Macromol. Biosci..

[74] Wu Y, Chen YX, Yan J, Quinn D, Dong P, Sawyer SW, Soman P (2016). Acta Biomater..

[75] Jeong JO, Park JS, Kim YA, Yang SJ, Jeong SI, Lee JY, Lim YM (2020). Polymers.

[76] Zhao L, Zhang H, Guo Z, Yu X, Jiao X, Li MH, Hu J, Appl ACS (2022). Mater. Inter..

[77] M. Suneetha, O.S. Moo, S.M. Choi, S.Zo, K.M. Rao, S.S. Han, Chem. Eng. J. **426**, 130847 (2021)

[78] Spencer AR, Primbetova A, Koppes AN, Koppes RA, Fenniri H, Annabi N, Biomater ACS (2018). Sci. Eng..

[79] Furlani F, Montanari M, Sangiorgi N, Saracino E, Campodoni E, Sanson A, Benfenati V, Tampieri A, Panseri S, Sandri M (2022). Biomater. Sci..

[80] Imani KBC, Jo A, Choi GM, Kim B, Chung JW, Lee HS, Yoon J (2022). Macromol. Rapid. Commun..

[81] Park K, Kang K, Kim J, Kim SD, Jin S, Shin M, Son D, Appl ACS (2022). Mater. Inter..

[82] Lee S, Park K, Kum J, An S, Yu KJ, Kim H, Shin M, Son D (2022). Polymers.

[83] Wu T, Cui C, Huang Y, Liu Y, Fan C, Han X, Yang Y, Xu Z, Liu B, Fan G, Liu W, Appl ACS (2020). Mater. Inter..

[84] Li Y, Gong Q, Han L, Liu X, Yang Y, Chen C, Qian C, Han Q (2022). Carbohyd. Polym..

[85] Shi MT, Dong RA, Hu J, Guo BL (2023). Chem. Eng. J..

[86] Yao B, Wang H, Zhou Q, Wu M, Zhang M, Li C, Shi G (2017). Adv. Mater..

[87] Liu Y, Liu J, Chen S, Lei T, Kim Y, Niu S, Wang H, Wang X, Foudeh AM, Tok JB, Bao Z (2019). Nat. Biomed. Eng..

[88] Lu B, Yuk H, Lin S, Jian N, Qu K, Xu J, Zhao X (2019). Nat. Commun..

[89] Yuk H, Lu B, Lin S, Qu K, Xu J, Luo J, Zhao X (2020). Nat. Commun..

[90] T. Cheng, F. Wang, Y.Z. Zhang, L.Li, S.Y. Gao, X.L. Yang, S. Wang, P.F. Chen, W.Y. Lai, Chem. Eng. J. **450**, 138311 (2022)

[91] Zhang S, Chen Y, Liu H, Wang Z, Ling H, Wang C, Ni J, Celebi-Saltik B, Wang X, Meng X, Kim HJ, Baidya A, Ahadian S, Ashammakhi N, Dokmeci MR, Travas-Sejdic J, Khademhosseini A (2020). Adv. Mater..

[92] Zheng Y, Wang YD, Zhang F, Zhang SM, Piatkevich KD, Zhou NJ, Pokorski JK (2022). Adv. Mater. Technol..

[93] Feig VR, Tran H, Lee M, Bao Z (2018). Nat. Commun..

[94] Feig VR, Tran H, Lee M, Liu K, Huang Z, Beker L, Mackanic DG, Bao Z (2019). Adv. Mater..

[95] Heo DN, Lee SJ, Timsina R, Qiu X, Castro NJ, Zhang LG (2019). Mat. Sci. Eng. C-Mater..

[96] Lopez-Larrea N, Criado-Gonzalez M, Dominguez-Alfaro A, Alegret N, Agua ID, Marchiori B, Mecerreyes D, Appl ACS (2022). Polym. Mater..

[97] Pan L, Yu G, Zhai D, Lee HR, Zhao W, Liu N, Wang H, Tee BC, Shi Y, Cui Y, Bao Z (2012). Proc. Natl. Acad. Sci. U. S. A..

[98] Shi Y, Pan LJ, Liu BR, Wang YQ, Cui Y, Bao ZA, Yu GH (2014). J. Mater. Chem. A.

[99] Li G, Huang K, Deng J, Guo M, Cai M, Zhang Y, Guo CF (2022). Adv. Mater..

[100] Shin M, Song KH, Burrell JC, Cullen DK, Burdick JA (2019). Adv. Sci..

[101] J. Park, N. Jeon, S. Lee, G. Choe, E. Lee, J. Y. Lee, Chem. Eng. J. **446**, 137344 (2022)

[102] Tringides CM, Boulingre M, Khalil A, Lungjangwa T, Jaenisch R, Mooney DJ (2023). Adv. Healthc. Mater..

[103] Meng XT, Yu XY, Lu YL, Pei Z, Wang GQ, Qi MR, Liu RR, Zhou JY, Guo XP, Zhou ZJ, Wang F (2023). J. Neural Eng..

[104] Park Y, Chung TS, Rogers JA (2021). Curr. Opin. Biotech..

[105] Huang ZL, Hao YF, Li Y, Hu HJ, Wang CH, Nomoto A, Pan TS, Gu Y, Chen YM, Zhang TJ, Li WX, Lei YS, Kim N, Wang CF, Zhang L, Ward JW, Maralani A, Li XS, Durstock MF, Pisano A, Lin Y, Xu S (2018). Nat. Electron..

[106] O'Brien CM, Holmes B, Faucett S, Zhang LG (2015). Tissue Eng. Part B Rev..

[107] F.A. Wei, T.J. Duan, L.G. Yao, W.G. Yang, J. Appl. Polym. Sci. **140**(7), e53468 (2022)

[108] Teo MY, RaviChandran N, Kim N, Kee S, Stuart L, Aw KC, Stringer J, Appl ACS (2019). Mater. Inter..

[109] X.H. Chen, Y.E. Wang, S. Zhang, J.S. Cui, X.Y. Ma, L.D. Tian, M.Y. Li, C.W. Bao, Q.H. Wei, B. Du, Polymer Testing **119**, 107905 (2023)

[110] Xie MJ, Gao Q, Fu JZ, Chen ZC, He Y (2020). Bio. Des. Manuf..

[111] Muller SJ, Mirzahossein E, Iftekhar EN, Bacher C, Schrufer S, Schubert DW, Fabry B, Gekle S (2020). PLoS ONE.

[112] Xu T, Gregory CA, Molnar P, Cui X, Jalota S, Bhaduri SB, Boland T (2006). Biomaterials.

[113] Kang TH, Lee SW, Hwang K, Shim W, Lee KY, Lim JA, Yu WR, Choi IS, Yi H, Appl ACS (2020). Mater. Inter..

[114] Choudhury D, Anand S, Naing MW (2018). Int. J. Bioprint..

[115] Jiang P, Yan C, Guo Y, Zhang X, Cai M, Jia X, Wang X, Zhou F (2019). Biomater. Sci..

[116] Rocha VG, Saiz E, Tirichenko IS, Garcia-Tunon E (2020). J. Mater. Chem. A.

[117] Bovone G, Guzzi EA, Bernhard S, Weber T, Dranseikiene D, Tibbitt MW (2022). Adv. Mater..

[118] Lai CW, Yu SS, Appl ACS (2020). Mater. Inter..

[119] Zhao WW, Chen LJ, Hu SM, Shi ZJ, Gao X, Silberschmidt VV (2020). Adv. Compos. Hybrid Mater..

[120] Zhao W, Cao J, Wang F, Tian F, Zheng W, Bao Y, Zhang K, Zhang Z, Yu J, Xu J, Liu X, Lu B (2022). Polymers.

[121] Fares MM, Radaydeh SK (2022). Polym. Compos..

[122] Hardman D, Hughes J, Thuruthel TG, Gilday K, Iida F, Robot IEEE (2021). Autom. Let..

[123] Kim S, Choi H, Son D, Shin M (2023). Gels.

[124] Shin S, Hyun J, Appl ACS (2022). Mater. Inter..

[125] Wu Q, Zhu FB, Wu ZL, Xie Y, Qian J, Yin J, Yang HY (2022). npj Flex. Electron..

[126] Distler T, Polley C, Shi F, Schneidereit D, Ashton MD, Friedrich O, Kolb JF, Hardy JG, Detsch R, Seitz H, Boccaccini AR (2021). Adv. Healthc. Mater..

[127] Park S, Shin BG, Jang S, Chung K, Appl ACS (2020). Mater. Inter..

[128] Dutta SD, Ganguly K, Randhawa A, Patil TV, Patel DK, Lim KT (2023). Biomaterials.

[129] Placone JK, Engler AJ (2018). Adv. Healthc. Mater..

[130] Janarthanan G, Lee S, Noh I (2021). Adv. Funct. Mater..

[131] Xie X, Xu Z, Yu X, Jiang H, Li H, Feng W (2023). Nat. Commun..

[132] Kiyotake EA, Thomas EE, Homburg HB, Milton CK, Smitherman AD, Donahue ND, Fung KM, Wilhelm S, Martin MD, Detamore MS, Biomed J (2022). Mater. Res. A.

[133] Abodurexiti A, Maimaitiyiming X (2022). Macromol. Chem. Phys..

[134] Shin W, Kim JS, Kim H, Choi HJ, Lee HJ, Um MK, Choi MK, Chung K (2021). Macromol. Mater. Eng..

[135] Z.Q. Guo, W.Y. Liu, A.M. Tang, Eur. Polym. J. **164**, 110977 (2022)

[136] F.Y. Hao, X. Maimaitiyiming, S. Sun, Macromol. Chem. Phys. **224**(2) 2200272 (2022)

[137] Lyu J, Zhou Q, Wang H, Xiao Q, Qiang Z, Li X, Wen J, Ye C, Zhu M (2023). Adv. Sci..

[138] Wei J, Xie J, Zhang P, Zou Z, Ping H, Wang W, Xie H, Shen JZ, Lei L, Fu Z, Appl ACS (2021). Mater. Inter..

[139] Zhao W, Huang B, Zhu L, Feng X, Xu J, Zhang H, Yan S (2022). Int. J. Biol. Macromol..

[140] Gallastegui A, Dominguez-Alfaro A, Lezama L, Alegret N, Prato M, Gomez ML, Mecerreyes D, Macro ACS (2022). Lett..

[141] Guo B, Zhong Y, Chen X, Yu S, Bai J (2023). Compos. Commun..

[142] Li ZQ, He XN, Cheng JX, Li HG, Zhang YF, Shi XJ, Yu K, Yang HY, Ge Q (2021). Int. J. Smart Nano Mater..

[143] Yan HH, Zhou J, Wang CY, Gong HQ, Liu W, Cen WH, Yuan GX, Long Y (2022). Smart Mater. and Struct..

[144] Odent J, Baleine N, Biard V, Dobashi Y, Vancaeyzeele C, Nguyen GTM, Madden JDW, Plesse C, Raquez JM (2022). Adv. Funct. Mater..

[145] Keate RL, Tropp J, Collins CP, Ware HOT, Petty AJ, Ameer GA, Sun C, Rivnay J (2022). Macromol. Biosci..

[146] Y. Hui, Y. Yao, Q.L. Qian, J.H. Luo, H.H. Chen, Z. Qiao, Y.T. Yu, L. Tao, N.J. Zhou, Nat. Electron. **5**, 893-903 (2022)

[147] Burke G, Devine DM, Major I (2020). Polymers.

[148] Y.R. Zhang, L.Chen, M.Z. Xie, Z.H. Zhan, D.S. Yang, P. Cheng, H.G. Duan, Q. Ge, Z.L. Wang, Mater. Today Phys. **27**, 100794 (2022)

[149] Quan H, Zhang T, Xu H, Luo S, Nie J, Zhu X (2020). Bioact. Mater..

[150] He Y, Yu R, Li X, Zhang M, Zhang Y, Yang X, Zhao X, Huang W (2021). ACS Appl. Mater. Inter..

[151] Zhang C, Zheng H, Sun J, Zhou Y, Xu W, Dai Y, Mo J, Wang Z (2022). Adv. Mater..

[152] Yin XY, Zhang Y, Cai XB, Guo QQ, Yang J, Wang ZL (2019). Mater. Horiz..

[153] Zhu H, Hu X, Liu B, Chen Z, Qu S, Appl ACS (2021). Mater. Inter..

[154] Ge Q, Chen Z, Cheng J, Zhang B, Zhang YF, Li H, He X, Yuan C, Liu J, Magdassi S, Qu S (2021). Sci. Adv..

[155] Rastin H, Zhang B, Bi J, Hassan K, Tung TT, Losic D (2020). J. Mater. Chem. B.

[156] Wu J, Yang R, Zhang L, Fan Z, Liu S (2015). Toxicol. Mech. Methods.

[157] Yang F, Jiang Q, Xie W, Zhang Y (2017). Chemosphere.

[158] XavierMendes A, MoraesSilva S, Connell CDO’, Duchi S, Quigley AF, Kapsa RMI, Moulton SE, Biomater ACS (2021). Sci. Eng..

[159] Serafin A, Murphy C, Rubio MC, Collins MN (2021). Mater. Sci. Eng. C-Mater..

[160] Zhang Y, Petibone D, Xu Y, Mahmood M, Karmakar A, Casciano D, Ali S, Biris AS (2014). Drug Metab. Rev..

[161] Shin SR, Jung SM, Zalabany M, Kim K, Zorlutuna P, Kim SB, Nikkhah M, Khabiry M, Azize M, Kong J, Wan KT, Palacios T, Dokmeci MR, Bae H, Tang XS, Khademhosseini A (2013). ACS Nano.

[162] Saleemi MA (2021). M Hosseini Fouladi, P V C Yong, K Chinna, N K Palanisamy, E H Wong. Chem. Res. Toxicol..

[163] W.J. Zhao, M. Zhou, L.Z. Lv, H.Q. Fu, J. Alloys Compd. **886**, 161083 (2021)

[164] Zheng S, Wang H, Das P, Zhang Y, Cao Y, Ma J, Liu SF, Wu ZS (2021). Adv. Mater..

[165] Boularaoui S, Shanti A, Lanotte M, Luo S, Bawazir S, Lee S, Christoforou N, Khan KA, Stefanini C, Biomater ACS (2021). Sci. Eng..

[166] Parvini E, Hajalilou A, Lopes PA, Tiago MSM, de Almeida AT, Tavakoli M (2022). Soft Matter.

[167] Fan L, Xiong Y, Fu Z, Xu D, Wang L, Chen Y, Xia H, Peng N, Ye S, Wang Y, Zhang L, Ye Q (2017). Mol. Med. Rep..

[168] Vijayavenkataraman S, Kannan S, Cao T, Fuh JYH, Sriram G, Lu WF (2019). Front. Bioeng. Biotechnol..

[169] Wang C, Rubakhin SS, Enright MJ, Sweedler JV, Nuzzo RG (2021). Adv. Funct. Mater..

[170] Liu J, Zhang B, Zhang P, Zhao K, Lu Z, Wei H, Zheng Z, Yang R, Yu Y (2022). ACS Nano.

[171] Serafin A, Rubio MC, Carsi M, Ortiz-Serna P, Sanchis MJ, Garg AK, Oliveira JM, Koffler J, Collins MN (2022). Biomater. Res..

[172] Aggas JR, Abasi S, Phipps JF, Podstawczyk DA, Guiseppi-Elie A (2020). Biosens. Bioelectron..

[173] Liu J, McKeon L, Garcia J, Pinilla S, Barwich S, Mobius M, Stamenov P, Coleman JN, Nicolosi V (2022). Adv. Mater..

[174] Lin CM, Lin JW, Chen YC, Shen HH, Wei L, Yeh YS, Chiang YH, Shih R, Chiu PL, Hung KS, Yang LY, Chiu WT (2009). Surg. Neurol..

[175] Taras JS, Jacoby SM, Lincoski CJ (2011). J Hand Surg. Am..

[176] X. Kuang, M.O. Arican, T. Zhou, X.H. Zhao, Y.S. Zhang, Acc. Mater. Res. **4**(2), 101-114 (2022)

[177] Lu C, Wang C, Yu J, Wang J, Chu F (2020). Chemsuschem.

[178] Do UT, Kim J, Luu QS, Nguyen QT, Jang T, Park Y, Shin H, Whiting N, Kang DK, Kwon JS, Lee Y (2023). Carbohyd. Polym..

[179] Helling AL, Tsekoura EK, Biggs M, Bayon Y, Pandit A, Zeugolis DI, Biomater ACS (2017). Sci. Eng..

[180] Fakhari A, Berkland C (2013). Acta Biomater..

[181] Ren D, Yi H, Wang W, Ma X (2005). Carbohydr. Res..

[182] Deshayes S, Kasko AM (2013). J. Polym. Sci. Pol. Chem..

[183] Engler AJ, Sen S, Sweeney HL, Discher DE (2006). Cell.

[184] Leipzig ND, Shoichet MS (2009). Biomaterials.

[185] Lantoine J, Grevesse T, Villers A, Delhaye G, Mestdagh C, Versaevel M, Mohammed D, Bruyere C, Alaimo L, Lacour SP, Ris L, Gabriele S (2016). Biomaterials.

[186] Cai J, Wang J, Sun C, Dai J, Zhang C (2022). Biomed. Mater..

[187] Zhu FB, Zheng SY, Lin J, Wu ZL, Yin J, Qian J, Qu SX, Zheng Q (2020). J. Mater. Chem. C.

[188] Ding XY, Jia RP, Gan ZZ, Du Y, Wang DY, Xu XW (2020). Mater. Res. Express.

[189] Wei H, Lei M, Zhang P, Leng J, Zheng Z, Yu Y (2021). Nat. Commun..

[190] Deng Z, Qian T, Hang F, Biomater ACS (2020). Sci. Eng..

[191] Liu CC, Zhao XL, Wang SP, Zhang YJ, Ge W, Li JJ, Cao J, Tao JY, Yang XW, Appl ACS (2019). Energ. Mater..

[192] Chen Z, Luo J, Hu Y, Fu Y, Meng J, Luo S, Wang L, Zhang Y, Zhou J, Zhang M, Qin H (2022). Int. J. Biol. Macromol..

[193] Seong M, Kondaveeti S, Choi G, Kim S, Kim J, Kang M, Jeong HE, Appl ACS (2023). Mater. Inter..

[194] Hu YJ, Zhuo H, Zhang Y, Lai HH, Yi JW, Chen ZH, Peng XW, Wang XH, Liu CF, Sun RC, Zhong LX (2021). Adv. Funct. Mater..

[195] Xu JP, Wong CW, Hsu SH (2020). Chem. Mater..

[196] Gong HY, Park J, Kim W, Kim J, Lee JY, Koh WG, Appl ACS (2019). Mater. Inter..

[197] Choi Y, Park K, Choi H, Son D, Shin M (2021). Polymers.

[198] Yu C, Schimelman J, Wang P, Miller KL, Ma X, You S, Guan J, Sun B, Zhu W, Chen S (2020). Chem. Rev..

[199] He Y, Li Q, Chen P, Duan Q, Zhan J, Cai X, Wang L, Hou H, Qiu X (2022). Nat. Commun..

[200] Vitali I, Fievre S, Telley L, Oberst P, Bariselli S, Frangeul L, Baumann N, McMahon JJ, Klingler E, Bocchi R, Kiss JZ, Bellone C, Silver DL, Jabaudon D (2018). Cell.

[201] C. Herrera-Rincon, V.P. Pai, K.M. Moran, J.M. Lemire, M. Levin, Nat. Commun.** 8**(1), 587 (2017) 10.1038/s41467-017-00597-2 PMC5610959 28943634

[202] Kuzmenko V, Karabulut E, Pernevik E, Enoksson P, Gatenholm P (2018). Carbohyd. Polym..

[203] Lee SJ, Zhu W, Nowicki M, Lee G, Heo DN, Kim J, Zuo YY, Zhang LG (2018). J. Neural Eng..

[204] Huang CT, Kumar Shrestha L, Ariga K, Hsu SH (2017). J. Mater. Chem. B.

[205] Rinoldi C, Lanzi M, Fiorelli R, Nakielski P, Zembrzycki K, Kowalewski T, Urbanek O, Grippo V, Jezierska-Wozniak K, Maksymowicz W, Camposeo A, Bilewicz R, Pisignano D, Sanai N, Pierini F (2021). Biomacromol.

[206] Bordoni M, Karabulut E, Kuzmenko V, Fantini V, Pansarasa O, Cereda C, Gatenholm P (2020). Cells.

[207] Wang J, Li X, Song Y, Su Q, Xiaohalati X, Yang W, Xu L, Cai B, Wang G, Wang Z, Wang L (2021). Bioact. Mater..

[208] Xu C, Chang YK, Wu P, Liu K, Dong XZ, Nie AM, Mu CP, Liu ZY, Dai HL, Luo ZQ (2021). Adv. Funct. Mater..

[209] Koffler J, Zhu W, Qu X, Platoshyn O, Dulin JN, Brock J, Graham L, Lu P, Sakamoto J, Marsala M, Chen S, Tuszynski MH (2019). Nat. Med..

[210] Tran KA, DeOre BJ, Ikejiani D, Means K, Paone LS, De Marchi L, Suprewicz L, Koziol K, Bouyer J, Byfield FJ, Jin Y, Georges P, Fischer I, Janmey PA, Galie PA (2023). Biomaterials.

[211] Qian Y, Zhao X, Han Q, Chen W, Li H, Yuan W (2018). Nat. Commun..

[212] Zhu W, Tringale KR, Woller SA, You S, Johnson S, Shen H, Schimelman J, Whitney M, Steinauer J, Xu W, Yaksh TL, Nguyen QT, Chen S (2018). Mater. Today.

[213] Dinis TM, Elia R, Vidal G, Dermigny Q, Denoeud C, Kaplan DL, Egles C, Marin F (2015). J Mech Behav. Biomed..

[214] Fang Y, Wang C, Liu Z, Ko J, Chen L, Zhang T, Xiong Z, Zhang L, Sun W (2023). Adv. Sci..

[215] Gao C, Li YX, Liu XY, Huang J, Zhang ZJ (2023). Chem. Eng. J..

[216] Liu X, Hao M, Chen Z, Zhang T, Huang J, Dai J, Zhang Z (2021). Biomaterials.

[217] Peng LH, Xu XH, Huang YF, Zhao XL, Zhao B, Cai SY, Xie MJ, Wang MZ, Yuan TJ, He Y, Xu Z, Gao JQ, Gao C (2020). Adv. Funct. Mater..

[218] Abodurexiti A, Maimaitiyiming X (2022). IEEE Sens. J..

[219] Krishnadoss V, Kanjilal B, Masoumi A, Banerjee A, Dehzangi I, Pezhouman A, Ardehali R, Martins-Green M, Leijten J, Noshadi I (2023). Mater. Today Adv..

[220] Han M, Yildiz E, Kaleli HN, Karaz S, Eren GO, Dogru-Yuksel IB, Senses E, Sahin A, Nizamoglu S (2022). Adv. Healthc. Mater..

[221] Wang C, Jiang X, Kim HJ, Zhang S, Zhou X, Chen Y, Ling H, Xue Y, Chen Z, Qu M, Ren L, Zhu J, Libanori A, Zhu Y, Kang H, Ahadian S, Dokmeci MR, Servati P, He X, Gu Z, Sun W, Khademhosseini A (2022). Biomaterials.

[222] Picchio ML, Gallastegui A, Casado N, Lopez-Larrea N, Marchiori B, del Agua I, Criado-Gonzalez M, Mantione D, Minari RJ, Mecerreyes D (2022). Adv. Mater. Technol..

[223] Zhou X, Rajeev A, Subramanian A, Li Y, Rossetti N, Natale G, Lodygensky GA, Cicoira F (2022). Acta Biomater..

[224] Yao BW, de Vasconcelos LS, Cui QY, Cardenas A, Yan YC, Du YJ, Wu D, Wu SW, Hsiai TK, Lu NS, Zhu XY, He XM (2022). Mater. Today.

[225] L. Chen, Z.L. Wang, Z.H. Zhan, M.Z. Xie, G.H. Duan, P. Cheng, Y.Q. Chen, H.G. Duan, Mater. Today Phys.** 19**, 100404 (2021)

[226] Wang Z, Chen L, Chen Y, Liu P, Duan H, Cheng P (2020). Research-China.

[227] Zhou T, Yuk H, Hu F, Wu J, Tian F, Roh H, Shen Z, Gu G, Xu J, Lu B, Zhao X (2023). Nat. Mater..

[228] Cao W, Peng S, Yao Y, Xie J, Li S, Tu C, Gao C (2022). Acta Biomater..

[229] Hiendlmeier L, Zurita F, Vogel J, Del Duca F, Al Boustani G, Peng H, Kopic I, Nikic M, Wolfrum B (2023). Adv. Mater..

[230] Li Z, Zhao Y, Wang Z, Ren M, Wang X, Liu H, Lin Q, Wang J (2022). Adv. Healthc. Mater..

[231] Ahn J, Sim HH, Kim JH, Wajahat M, Kim JH, Bae J, Kim S, Pyo J, Jeon CJ, Kim BS, Baek SH, Seol SK (2021). Adv. Mater. Technologies.

[232] Da Silva AC, Wang J, Minev IR (2022). Nat. Commun..

[233] Lee K, Park M, Malollari KG, Shin J, Winkler SM, Zheng YT, Park JH, Grigoropoulos CP, Messersmith PB (2020). Nat. Commun..

[234] R. Miyakoshi, S. Hayashi, M. Terakawa, Adv. Electron. Mater. **9**(5), 2201277 (2023)

